# The metastatic promoter DEPDC1B induces epithelial‐mesenchymal transition and promotes prostate cancer cell proliferation via Rac1‐PAK1 signaling

**DOI:** 10.1002/ctm2.191

**Published:** 2020-10-06

**Authors:** Zean Li, Qiong Wang, Shirong Peng, Kai Yao, Junxiu Chen, Yiran Tao, Ze Gao, Fen Wang, Hui Li, Wenli Cai, Yiming Lai, Kaiwen Li, Xu Chen, Hai Huang

**Affiliations:** ^1^ Department of Urology Sun Yat‐sen Memorial Hospital, Sun Yat‐sen University Guangzhou Guangdong P. R. China; ^2^ Guangdong Provincial Key Laboratory of Malignant Tumor Epigenetics and Gene Regulation Sun Yat‐Sen Memorial Hospital Sun Yat‐Sen University Guangzhou Guangdong P. R. China; ^3^ Department of Urology Sun Yat‐sen University Cancer Center Guangzhou Guangdong P. R. China; ^4^ Center for Cancer and Stem Cell Biology Texas A&M Health Science Center Institute of Biosciences and Technology Houston Texas; ^5^ Department of Pathology School of Medicine University of Virginia Charlottesville Virginia; ^6^ Department of Radiology Massachusetts General Hospital Harvard Medical School Boston Massachusetts

**Keywords:** DEPDC1B, EMT, prostate cancer, Rac1

## Abstract

Metastasis is the major cause of prostate cancer (PCa)‐related mortality. Epithelial‐mesenchymal transition (EMT) is a vital characteristic feature that empowers cancer cells to adapt and survive at the beginning of metastasis. Therefore, it is essential to identify the regulatory mechanism of EMT in metastatic prostate cancer (mPCa) and to develop a novel therapy to block PCa metastasis. Here, we discovered a novel PCa metastasis oncogene, DEP domain containing 1B (DEPDC1B), which was positively correlated with the metastasis status, high Gleason score, advanced tumor stage, and poor prognosis. Functional assays revealed that DEPDC1B enhanced the migration, invasion, and proliferation of PCa cells in vitro and promoted tumor metastasis and growth in vivo. Mechanistic investigations clarified that DEPDC1B induced EMT and enhanced proliferation by binding to Rac1 and enhancing the Rac1‐PAK1 pathway. This DEPDC1B‐mediated oncogenic effect was reversed by a Rac1‐GTP inhibitor or Rac1 knockdown. In conclusion, we discover that the DEPDC1B‐Rac1‐PAK1 signaling pathway may serve as a multipotent target for clinical intervention in mPCa.

## Graphical abstract

Highlights

1. DEPDC1B positively correlates with prostate cancer metastasis, tumor stage, Gleason score, and poor prognosis.

2. DEPDC1B enhances prostate cancer cell metastasis and tumor growth in vitro and in vivo.

3. DEPDC1B enhances the Rac1‐PAK1 signaling pathway to induce EMT.

4. DEPDC1B contributing to metastasis and proliferation through Rac1‐PAK1 signaling.

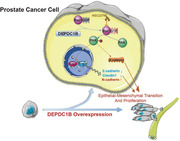

**Abbreviations**
(m)PCa: (metastatic) prostate cancer
BCR‐free survival: biochemical recurrence‐free survival
co‐IP: coimmunoprecipitation
DEPDC1B: DEP domain containing 1B
DFS: disease‐free survival
EMT: epithelial‐mesenchymal transition
GAPs: GTPase‐activating proteins
GDIs: guanine nucleotide dissociation inhibitors
GEFs: guanine nucleotide exchange factors
GEO: gene expression omnibus
GPCR: G protein‐coupled receptor
IHC: immunohistochemistry
MS: mass spectrometry
NSCLC: non‐small‐cell lung cancer
OS: overall survival
PPI: protein‐protein interaction
PSA: prostate‐specific antigen
qRT‐PCR: quantitative reverse transcriptase‐polymerase chain reaction
siRNA: RNA interference
TCGA: The Cancer Genome Atlas
TMA: tissue microarray
TURP: transurethral resection prostate

## 1 INTRODUCTION

Prostate cancer (PCa) is the second most commonly diagnosed malignant tumor in men, with 1.3 million new cases (13.5% of total cancer cases in males) worldwide.^1^ The mortality of metastatic PCa (mPCa) is significantly higher than that of nonmetastatic PCa, and the death rate increases to 71% within 5 years even if patients undergo radical prostatectomy.^1^, ^2^, ^3^, ^4^ Metastasis is a complex process involving the escape of cancer cells from the primary tumor into the blood vessels or lymph channels, survival in the circulation, arrest and extravasation into the secondary site, and initiation and maintenance of growth to form clinically detectable metastases.^5^ Epithelial‐mesenchymal transition (EMT), an essential process for cancer metastasis, enables cancer cells transform from polarized, differentiated, and epithelial‐like cells into isolated, undifferentiated, and mesenchymal‐like cells. Then, the tumor acquires stronger migratory and invasive capabilities to metastasize.^5^ Therefore, it is important to explore the mechanism of PCa metastasis and identify a novel biomarker and treatment target for mPCa.

As far as current research is concerned, multiple signaling pathways, including wnt/β‐catenin, Rho GTPase, PI3K, and MAPK, which reprogram the cytoskeleton and regulate cell‐cell adhesion, are involved in cancer metastasis.^5^, ^6^, ^7^ The Rho GTPase family, which consists of several members, such as Rac1, cdc42, and Rnd1, belongs to the Ras superfamily. In addition to modulating tumor metastasis, the Rho family is mainly involved in the regulation of cell shape, differentiation, and proliferation.^8^ Guanine nucleotide exchange factors (GEFs), GTPase‐activating proteins (GAPs), or guanine nucleotide dissociation inhibitors (GDIs) are required for modulating the functions of Rho family members.^8^ The GTPase Rac1 is a well‐established master regulator of cell motility and invasiveness contributing to cancer metastasis. Dysregulation of the Rac1 signaling pathway, resulting in elevated motile and invasive potential, has been reported in PCa. Recent research showed that P‐REX1 and Vav3 were served as the regulator of Rac1 in modulating the mobility of PCa.^9^, ^10^ In addition, RhoH, a regulator of Rac1, which depleted, is able to reduce lamellipodium extension by regulating the localization of Rac1.^11^ However, Rho signaling network is complex, and the detail mechanism of Rac1 in mPCa remains largely unknown.

DEPDC1B is a gene localized at human chromosome 5q12.1.^12^ It contains an inactive RhoGAP domain which lacks a critical arginine residue to induce GAP activity,^12^, ^13^ and a DEP domain which is responsible for specific recognition of G protein‐coupled receptor (GPCR).^14^, ^15^ In a previous study, DEPDC1B was found to be a protein involving in the cell cycle and mitotic entry. Knockdown of DEPDC1B arrested the cell cycle at G2/M and retarded entry into mitosis.^16^ DEPDC1B also participates in the formation and dismantling of focal adhesions.^17^ Yang et al found that DEPDC1B is overexpressed in non‐small‐cell lung cancer (NSCLC) and promotes the migration ability of NSCLC cells via wnt/β‐catenin.^12^ Additionally, Ying‐Fang Su et al indicated that DEPDC1B can enhance anchorage‐independent growth and migration by activating the ERK signaling pathway.^13^In our previous study, we found that increased expression of DEPDC1B was associated with LN metastasis and poor prognose of PCa.^18^ However, the detail biological function and mechanism of DEPDC1B in PCa remained largely unknown.

In our present study, DEPDC1B was overexpressed in mPCa patients, and a high level of DEPDC1B positively correlated with a high Gleason score and poor prognosis. We also found that DEPDC1B binds with Rac1 and then increases the Rac1‐GTP level to enhance the Rac1‐PAK1 signaling pathway, which can induce EMT and ultimately enhance wnt/β‐catenin signaling to promote PCa metastasis and progression.

## 2 MATERIALS AND METHODS

### 2.1 Data analysis of GEO and TCGA databases

Three datasets (GSE3325,^19^ GSE6919,^20^ and GSE67872^21^) analyzed during the current study are available in the Gene Expression Omnibus (GEO) (https://www.ncbi.nlm.nih.gov/geo/). The three datasets were based on the GPL570, GPL92, and GPL10361 platform, respectively. The GEO2R tool was used to analyze the GSE3325 dataset (including six normal samples, seven localized PCa samples, and six mPCa samples), the GSE6919 (including 77 normal samples, 66 localized PCa samples, and 25 mPCa samples), and GSE67872 (including three normal samples and three mPCa samples from mice). Merely genes from GSE3325 and GSE67872 with | log_2_fold change | > 2 and *P* < .05 and genes from GSE6919 with | log_2_fold change | > 1 and *P* < .05 were selected to further investigation.

The gene expression data of DEPDC1B and the clinical information of The Cancer Genome Atlas (TCGA), including 489 PCa samples and 51 adjacent normal prostate tissue samples, were collected to analyze the clinical variables and perform survival analysis. The cases without survival data were excluded for analysis. The PCa samples were classified as low or high level of DEPDC1B expression by using the median.

### 2.2 Human tissue samples

A tissue microarray (TMA; n = 192; catalog no. PR1921c) cohort (Cohort 1), including 160 PCa tissue samples and 32 adjacent or normal prostate tissue samples, was obtained from Alenabio Biotechnology, Ltd. (Xi'an, China), a distributor of US Biomax, Inc. (Rockville, MD, USA) in China.

A total of 103 paraffin‐embedded PCa tissues (Cohort 2) were obtained with the written consent of patients who underwent radical prostatectomy or transurethral resection prostate (TURP) at Sun Yat‐sen University Cancer Center (Guangzhou, China) between January 2000 and August 2018. All samples were diagnosed with PCa by two independent pathologists. TNM stage and Gleason score of PCa samples were confirmed according to the guidelines. Ethical approval was obtained from Sun Yat‐sen University's Committees for Ethical Review of Research Involving Human Subjects. All patients were followed up until December 2018.

### 2.3 IHC staining and scoring analyses

Paraffin sections of PCa tissues and TMA were first deparaffinized and hydrated. All antigens were retrieved by the microwave method, and endogenous peroxidase activity was blocked by incubating the slides in 0.3% H_2_O_2_. After incubation with the primary and secondary antibodies, sections were developed with peroxidase and 3,3′‐diaminobenzidine tetrahydrochloride. The sections were then counterstained with hematoxylin and mounted in nonaqueous mounting medium. An anti‐DEPDC1B antibody (1:20; HPA072558; Atlas Antibodies, Stockholm, Sweden) was used to detect DEPDC1B expression in the specimens and mouse tumors. Antibodies against Ki67 (1:500; Servicebio; Wuhan, China), E‐cadherin (1:200, Cell Signaling Technology, Inc, Danvers, MA, USA), and N‐cadherin (1:200, Cell Signaling Technology) were used to detect Ki67, E‐cadherin, and N‐cadherin expression, respectively, in mouse tumors.

DEPDC1B expression in the PCa samples was blind quantified by two pathologists. The immunostaining intensity of each sample was divided into negative = 0, weak = 1, moderate = 2, or strong = 3. The proportion of positively stained cells was graded to <25% = 1, 25‐50% = 2, 50‐75% = 3, and >75% = 4. The score was then calculated as the intensity score multiplied by the proportion score (score = intensity × proportion score). The samples were classified as low (score ≤6) or high (score > 6) DEPDC1B expression. Images were visualized using a Nikon Eclipse Ni‐U (Nikon, Japan) microscope system and processed with NIS‐Elements software.

### 2.4 Cell culture

The human PCa cell lines (DU145 and PC3) and the SV40‐transformed kidney cell line 293T were purchased from ATCC (American Type Culture Collection, Manassas, VA, USA). DU145 and 293T cells were cultured in DMEM (Gibco, Shanghai, China), whereas PC3 cells were cultured in RPMI 1640 (Gibco). All cell culture medium was supplemented with 10% FBS and 1% penicillin/streptomycin (Gibco). The Rac1‐GTP inhibitor NSC23766 (Cat. No. S8031) was purchased from Selleck (Houston, TX, USA) and used at 30 µM for 48 h. Cells were cultured in a humidified atmosphere of 5% CO_2_ at 37°C (BB150, Thermo Scientific, Shanghai, China).

### 2.5 RNA isolation and qRT‐PCR

Total RNA was isolated using RNAiso Plus (9109, TaKaRa, Japan) according to the manufacturer's instructions. The cDNA was synthesized using the PrimeScript RT Reagent Kit (RR047A, TaKaRa, China). The expression of mRNA in prostate cell lines was determined with an ABI QuantStudio Sequence Detection System (Applied Biosystems). Each reaction was performed in triplicate. The specific primers are listed in Supporting information Table S1.

### 2.6 Transient transfection

RNA interference (siRNA) oligonucleotides targeting DEPDC1B, Rac1, and negative control siRNAs were purchased from GenePharma (Shanghai, China). The siRNA sequences are listed in Supporting information Table S1. siRNA transfections were performed using 75 nM siRNA with 3 µL/mL Lipofectamine RNAimax (Life Technologies, Waltham, MA, USA) and incubated for 48 h for RNA isolation and 72 h for protein collection.

The pcDNA3.1 Rac1(Q61L) (#13720, Addgene, Watertown, USA) or an empty vector was transiently transfected into prostate cells with X‐tremeGENE HP DNA Transfection Reagent (6366546001, Roche, Basel, Switzerland) and cultured for 72 h for further investigation.

### 2.7 Western blotting

Western blotting was performed as previously described.^22^ Primary antibodies specific to DEPDC1B (1:500, ab124182, Abcam), Rac1 (1:1000, ab33186, Abcam), pPAK1 (2605S), N‐cadherin (13116S), E‐cadherin (3195S), snail (3879S), slug (9585S), claudin1 (13255), β‐catenin (9562, 1:1000, Cell Signaling Technology), PAK (A0809, ABclonal Biotechnology, Wuhan, China), and actin (AA128, Beyotime, Shanghai, China) were used. The blots were then incubated with a goat antirabbit (cw0103s) or antimouse secondary antibody (cw0102s, 1:10,000, Cwbiotech, Beijing, China), and the blots were visualized using Immobilon Western Chemiluminescent HRP Substrate (WBKLS0500, Merck Millipore, Germany).

### 2.8 Coimmunoprecipitation (Co‐IP) assays

Co‐IP assays of DEPDC1B and Rac1 were performed according to the manufacturer's instructions of the Pierce Crosslink Magnetic IP/Co‐IP Kit (88805, Thermo Scientific). Briefly, 2 µg of an anti‐Flag (14793), anti‐IgG(3900, CST), or anti‐Rac1(ab33186, Abcam) antibody was bound to Protein A/G Magnetic Beads, and the antibody was covalently cross‐linked to the beads using disuccinimidyl suberate (DSS). The antibody cross‐linked beads were then incubated overnight at 4°C with 400 µg cell lysate that contained Flag‐DEPDC1B and Rac1. The beads were washed to remove unbound proteins, and a low pH elution buffer was used to dissociate bound antigen from the antibody cross‐linked beads. Neutralization buffer was included to prevent precipitation of the isolated antigen and to ensure protein activity in downstream applications. Lane Marker Sample Buffer was used to prepare samples for SDS‐PAGE immunoblot analysis with specific antibodies against or the SDS‐PAGE gel was silver stained by using the Pierce Silver Stain Kit (24612, Thermo Scientific). The proteins eluted from magnetic beads were followed by mass spectrometry (MS) analysis with an Easy nanoLC 1200‐Orbitrap Fusion (Thermo Fisher, USA), and the proteins identified by MS were analyzed on the Metascape website (http://metascape.org) and the String website (https://string-db.org).

### 2.9 Active Rac1 pull‐down assay

The RAC1 pull‐down assay was constructed according to the manufacturer's instructions for the Rac1 Activation Assay Biochem Kit (BK035, Cytoskeleton, Denver, USA). Briefly, cell lysates were collected for Rac1 activation studies when the cells had grown to approximately 70% confluence. Then, 400 µg protein lysate was added to a predetermined 10 µg amount of PAK‐PBD beads. The mixture was incubated at 4°C on a rotator for 1 h. The supernatant was carefully removed after the PAK‐PBD beads were pelleted by centrifugation, and the beads were washed once with 500 µL of wash buffer. Then, 10‐20 µL of 2× Laemmli sample buffer was added to each tube, and the bead samples were boiled for 2 min. Finally, the samples were analyzed by SDS‐PAGE and Western blot analyses with an anti‐Rac1 antibody.

### 2.10 Lentivirus transduction

To establish stable knockdown and overexpression prostate cell lines, full‐length DEPDC1B or shRNA sequences that specifically target DEPDC1B were cloned into the vectors pCDH‐GFP‐CMV‐EF1‐Puro‐3xFlag or pLKO.1‐Puro. The sequences of the shRNAs are listed in Supporting information Table S3. Lentivirus production and infection were conducted as described previously.^23^

### 2.11 Cell proliferation assay

The MTS assay and the colony formation assay were performed to detect cell viability. PCa cells (1000 DU145 cells or 1500 PC3 cells per well) were seeded into 96‐well plates, and 20 µL MTS (Promega, Beijing, China) was added to each well for a 2 h incubation. Then, we measured the absorbance of each well at 492 nm every 24 h six times. The same cells were seeded into 6‐well plates and cultured for 10 days for the colony formation assay.

### 2.12 Cell migration and invasion assay

A migration assay was performed by filling the bottom well of the cell culture insert (353097, Corning, NY, USA) with DMEM or RPMI 1640 medium containing 10% FBS. The insert wells were covered with polyethylene terephthalate (PET) membranes with 8‐µm pores, and 40 000 cells/well in serum‐free DMEM or RPMI 1640 were added to the top culture insert. The cell culture insert chamber was incubated for 24 h at 37°C to allow the possible migration of cells through the membrane into the bottom chamber. Membranes were stained using crystal violet. The cells in the bottom chamber were counted using an Olympus IX71 inverted microscope (Olympus, Japan). The PET membranes were covered with Matrigel Basement Membrane Matrix (354234, Corning) for the cell invasion assay.

### 2.13 3D cell migration model

The 3D cell migration model was built using Matrigel Basement Membrane Matrix (354234, c). One hundred microliters of Matrigel was placed flat on the 24‐well plate, and the cell suspension (3000/well) was mixed with the gel (vol:vol = 1:1) after incubation for 1 h at 37°C. Subsequently, the culture medium was added to the plate after 24 h and cultured for 72 h until we observed it under a Nikon Eclipse Ni‐U upright microscope (Nikon, Tokyo, Japan).

### 2.14 In vivo metastasis and tumorigenesis experiments

The in vivo metastasis assay was performed as previously described.^24^, ^25^ All procedures involving animals were approved by the Institute Animal Care and Use Committee of Sun Yat‐sen University. Male BALB/c nude mice (4‐6 weeks old) were purchased from the Experimental Animal Center of Sun Yat‐sen University and housed in specific pathogen‐free (SPF) barrier facilities. Five mice were included in each group, and lentivirus‐transduced PC3 cells (5 × 10^6^ cells) that stably expressed firefly luciferase were injected into the mouse footpads. Lymphatic metastasis was monitored and imaged using a bioluminescence imaging system (Cypris FIS‐250D [Xupu, China]). Eight weeks after the injections, the mice were euthanized, and the tumors were surgically dissected. The popliteal lymph nodes (LNs) were embedded in paraffin. The formalin‐fixed, paraffin‐embedded (FFPE) samples were analyzed using hematoxylin‐eosin (H&E) staining. Images were captured using a Nikon Eclipse Ni‐U system with NIS‐Elements software (Nikon, Tokyo, Japan)

A total of 3 × 10^6^ cells were injected subcutaneously into the right side of the dorsum, and five mice were used in each group. At 6 weeks after implantation, the mice were euthanized, and the tumors were surgically dissected. The tumor specimens were fixed in 4% paraformaldehyde. The volumes of the tumor were calculated using the following formula: tumor volume (mm^3^) = (length [mm]) × (width [mm])^2^ × 0.5. The weight of the tumor was also recorded.

### 2.15 Statistical analyses

Quantitative data are presented as the means ± SDs of three independent experiments. Differences between two groups were analyzed using the unpaired *t*‐test (two‐tailed tests). One‐way ANOVA followed by Dunnett's multiple comparison test was performed to compare more than two groups.^26^ Two‐way ANOVA was performed to analyze the factorial design to investigate the relationship between DEPDC1B and Rac1.

All primary data in the TAM and clinical samples were analyzed. Pearson's χ^2^ test was used to analyze the clinical variables. Cumulative survival time was calculated using the Kaplan‐Meier method and analyzed by the log‐rank test. All statistical analyses in this study were performed using SPSS 20.0 software (SPSS, Armonk, NY, USA) or GraphPad Prism 5.0 (GraphPad, La Jolla, CA, USA). A *P*‐value < .05 was considered significant.

## 3 RESULTS

### 3.1 Database‐integrated screening identifies that DEPDC1B correlates with the PCa metastasis status

**FIGURE 1:**
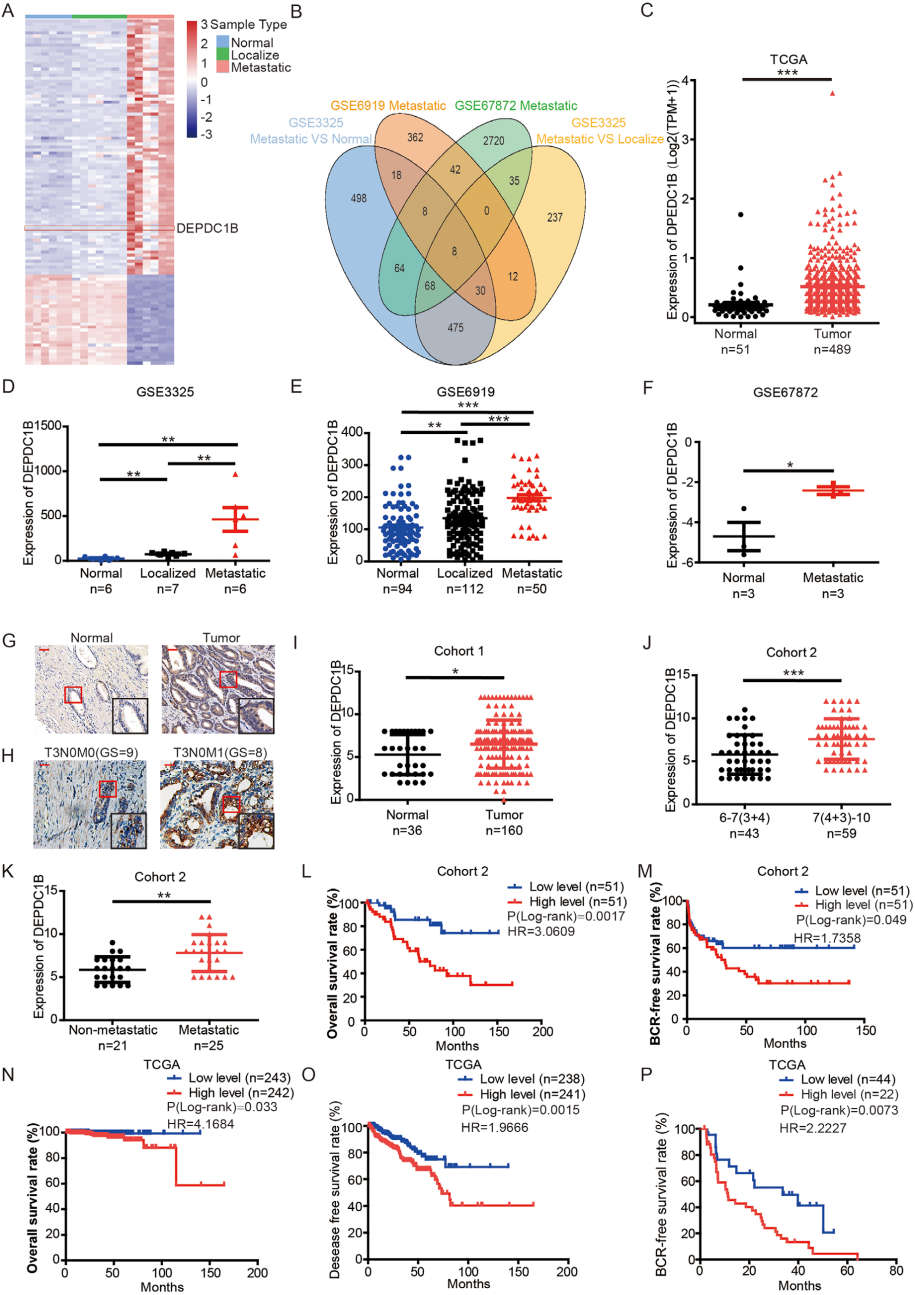
DEPDC1B expression is correlated with metastasis and poor prognosis of PCa. (A) The heatmap and unsupervised hierarchical clustering of mRNA expressed in mPCa, localized PCa and normal tissues in the GSE3325 dataset (fold change > 2.0, *P* < .05). The red color scale represents a higher expression level, and the blue color scale represents a lower expression level. (B) Venn diagram shows the overlapping genes with high expression in mPCa among three GEO datasets, including GSE3325 (mPCa vs. localized PCa and mPCa vs. normal), GSE6919 (mPCa vs. normal, fold change > 2.0, *P* < .05), and GSE67872 (mPCa vs. normal, fold change > 1.5, *P* < .05). (C) The expression of DEPDC1B in TCGA database. (D‐F) The expression of DEPDC1B in comparison among normal tissue, localized PCa, and mPCa in GSE3325 (D) and GSE6919 (E), and that in comparison between normal tissue with mPCa in the GSE67872 dataset (F). (G) Representative images of DEPDC1B expression in tumor tissues and normal tissues. (H) Representative images of DEPDC1B expression in mPCa (T3N0M1) and non‐mPCa (T3N0M0). (I‐K)The expression of DEPDC1B in Cohort 1 (I) that compared normal tissue with PCa, and in Cohort 2 that compared Gleason scores 6–7(3+4) with Gleason scores 7(4+3)‐10 (J), and compared non‐mPCa with mPCa (K). (L and M) Kaplan‐Meier curves for OS (L), BCR‐free survival (M) of PCa patients with high versus low expression of DEPDC1B in Cohort 2. (N‐P) Kaplan‐Meier curves for OS (N), DFS (O), and BCR‐free survival (P) of PCa patients with high versus low expression of DEPDC1B in TCGA. *P < .05, **P < .01, and ***P<.001,Scale bars: 50 µm

To identify potential mPCa oncogenes that could serve as targets for mPCa treatment, we performed a comprehensive analysis to screen data sets correlated with mPCa in the GEO database. First, we compared the expression levels of genes among normal, localized, and metastatic samples of the GSE3325 dataset and identified 581 metastasis‐related genes (Figure 1A). Similarly, we then identified 480 and 2945 metastasis‐related genes in the GSE6919 and GSE67872 databases, respectively. Furthermore, the genes that were overexpressed in mPCa samples from three datasets were integrated, and eight genes were selected (Figure 1B). To further identify a functional oncogene involved in mPCa, we analyzed TCGA database and found that four of these eight genes, DEPDC1B, CEP55, GAS2L3, and PLXNA1, were correlated with the prognosis of PCa and were significantly overexpressed in a malignancy (Figure 1C). However, Kaplan‐Meier survival analysis of TCGA database showed that high expression of CEP55, GAS2L, or PLXNA1 was only correlated with poor prognosis in disease‐free survival (DFS) but not correlated with overall survival (OS) (Supporting information Figure S1). Only DEPDC1B correlated with poor prognosis both in DFS and OS (Figure 1N‐P). In our previous study, we found that increased expression of DEPDC1B was associated with LN metastasis of PCa.^18^ However, the mechanism of how DEPDC1B regulates mPCa remains unclear. Then, DEPDC1B was selected for further investigation. Moreover, DEPDC1B showed a higher expression both in metastatic samples of PCa patients and metastatic mouse model than normal prostate tissue (Figure 1D‐F). More importantly, the expression level of DEPDC1B was higher in mPCa than in localized PCa (Figure 1D and E). These results demonstrated that DEPDC1B positively correlated with the metastatic status of PCa.

### 3.2 DEPDC1B expression is correlated with metastasis and poor prognosis of PCa

To further investigate whether DEPDC1B was involved in clinical PCa progression at the protein level, we analyzed a TMA cohort (Cohort 1) consisting of 192 samples that included normal, primary tumors, and LN metastases and a large‐scale sample cohort (Cohort 2) containing 103 PCa specimens. We found that the expression of DEPDC1B was significantly higher in primary tumors than in normal tissues (Figure 1G and I), while DEPDC1B expression was associated with a higher Gleason score in Cohort 1 (Supporting information Table S2).

Moreover, the expression of DEPDC1B was significantly upregulated in mPCa compared with nonmetastatic tumors (Figure 1H and J). The analysis of Cohort 2 also demonstrated that a high level of DEPDC1B positively correlated with a high Gleason score (*P* = .002), advanced pathological tumor stage (*P*<.001), and a high incidence of LN metastasis (*P* = .022; Figure 1J, Table 1). These results were further confirmed by analyses of the TCGA database, and we also found that a high level of DEPDC1B correlated with distant metastasis (*P* = .017; Supporting information Table S3).

**TABLE 1:** Associations between DEPDC1B expression and clinicopathological characteristics of PCa patients in Cohort 2

Clinical feature	Total patients n.	Low n.	High n.	*P*‐value
Age				
≤65	45	21	24	1
>65	57	27	30	
Gleason score				
≤6	29	20	9	.002**
3 + 4	14	9	5	
4 + 3	12	6	6	
≥8	47	13	34	
T state				
T1‐2	20	15	5	.033*
T3‐4	50	22	28	
Lymph node metastasis				
N0	41	26	15	.032*
N1	30	11	19	
Distant metastasis				
M0	21	14	7	.017*
M1	25	7	18	

A P‐value < .05 was considered significant. *P < .05, **P < .01, and ***P < .001

Furthermore, Kaplan‐Meier survival analysis showed that high expression of DEPDC1B was correlated with poor prognosis in not only OS but also biochemical recurrence (BCR)‐free survival (Figure 1L and M ). Consistent with our data, the TCGA database revealed that patients with high DEPDC1B‐expressing PCa had significantly shorter OS, BCR‐free survival, and DFS times (Figure 1N‐P). All the results demonstrated that DEPDC1B was associated with the metastasis and progression of PCa.

### 3.3 DEPDC1B promotes PCa cell metastasis and proliferation in vitro

**FIGURE 2:**
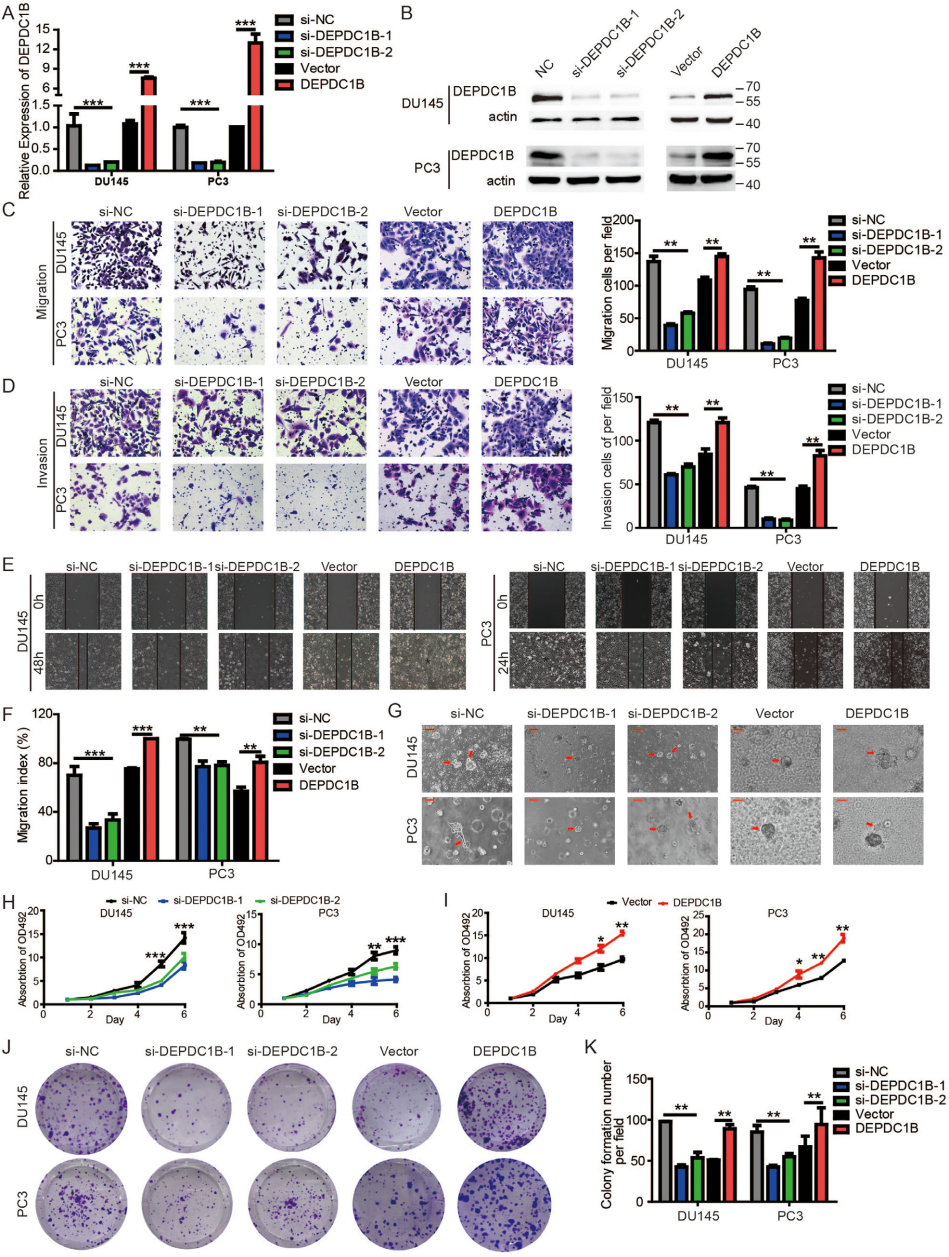
DEPDC1B promotes PCa cell metastasis and progression in vitro. (A and B) The qRT‐PCR (A) and Western blot (B) analysis of DEPDC1B expression levels in DEPDC1B‐knockdown, ‐overexpressing cells, and control cells. (C and D) Representative images of migration (C) and invasion (D) assays using DU145 and PC3 cells (left panels) and a quantification analysis of migrated or invaded cell counts (right panels), showing cell migration and invasion after downregulation or upregulation of DEPDC1B. (E) Representative images of wound‐healing assays using DU145 and PC3 cells, showing cell motility after downregulation or upregulation of DEPDC1B. (F) A quantification analysis of the cell migration index is shown. (G) Representative images of three‐dimensional (3D) cell culture using DU145 and PC3 cells, showing the cell invasive ability in stereoscopic space. (H and I) Cell viability was evaluated in DEPDC1B‐knockdown (H) or ‐overexpressing (I) DU145 and PC3 cells. (J) Colony formation assays were constructed in DEPDC1B‐knockdown or ‐overexpressing DU145 and PC3 cells. (K) A quantification analysis of colony formation number was shown. *P < .05, **P < .01, and ***P<.001. Scale bars: 25 µm

To investigate the function of DEPDC1B in the metastasis of PCa, DU145 and PC3 cell lines were transfected with two small interfering RNAs (siRNAs) targeting DEPDC1B or were established to stably overexpress DEPDC1B by lentiviral transfection. The efficiency of DEPDC1B‐knockdown and ‐overexpression was verified by qRT‐PCR and Western blotting. The results showed that the mRNA and protein levels of DEPDC1B were significantly downregulated or increased in DU145 and PC3 cell lines (Figure 2A and B). Then, we performed cell migration, invasion, wound‐healing assays, and three‐dimensional (3D) cell culture to explore the role of DEPDC1B in regulating motility and metastasis in PCa cells. Cell migration and invasion assays revealed that knockdown of DEPDC1B reduced the migration and invasion cell numbers, while the opposite outcome was found after overexpressing DEPDC1B (Figure 2C and D). Wound‐healing assays showed that silencing DEPDC1B decreased, whereas upregulating DEPDC1B increased, the migratory speed of DU145 and PC3 cells (Figure 2E and F). Moreover, DU145 and PC3 cells were embedded in Matrigel for 3D culture, which mimics the process of tumor invasion in the basement membrane, and we also found that DEPDC1B ablation inhibited the invasive capability, while overexpressing DEPDC1B accelerated invasion of PCa cell (Figure 2G). Similar results were found in stable DEPD1B‐knockdown PCa cell lines (Supporting information Figure S2A‐S2I). Taken together, our results demonstrated that DEPDC1B played a crucial role in PCa migration and invasion.

After detaching from the primary tumor, metastatic cells should gain the significant ability of proliferation for tumorigenesis when they settle at distal sites. Therefore, we performed cell proliferation assays and colony formation assays to investigate the function of DEPDC1B in proliferation. The results showed that silencing DEPDC1B reduced the cell viability and colony formation ability of DU145 and PC3 cells, while overexpression of DEPDC1B enhanced the viability and colony formation ability of PCa cells (Figure 2H‐K). The stable DEPDC1B‐knockdown PCa cell lines showed analogous results (Supporting information Figure S2J‐S2L). These results indicated that DEPDC1B was required for PCa metastasis and proliferation.

### 3.4 DEPDC1B facilitates metastasis and tumor growth of PCa cells in vivo

**FIGURE 3:**
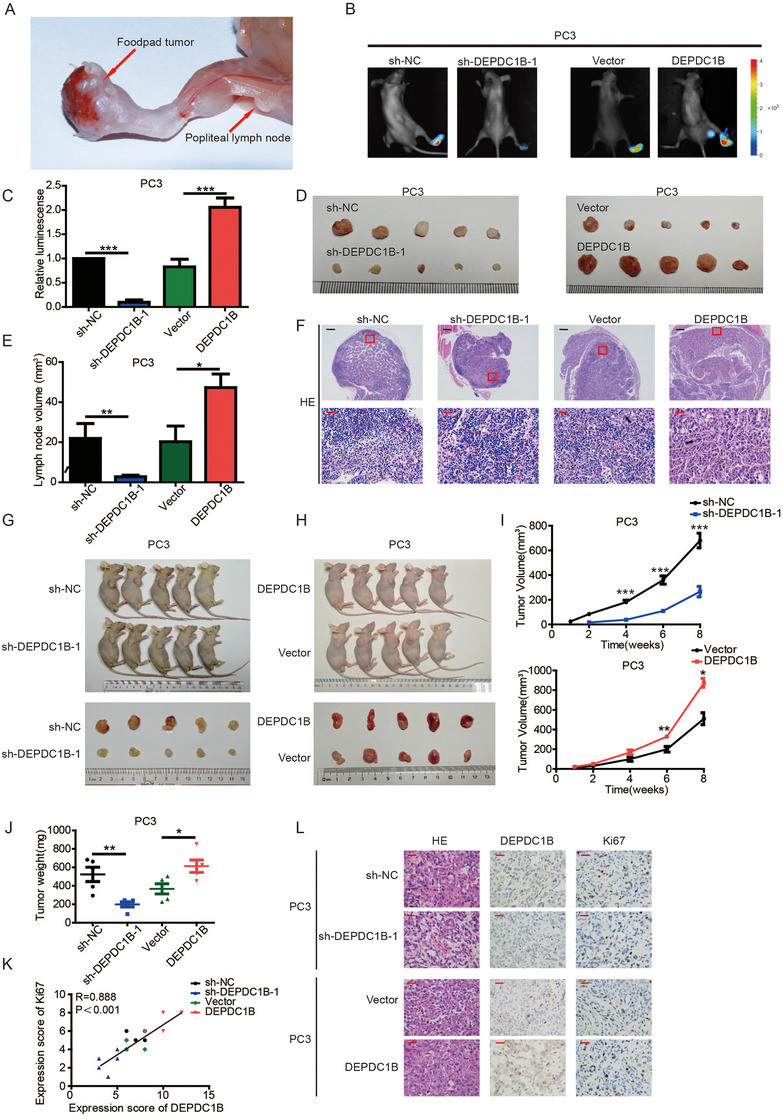
DEPDC1B facilitates the metastasis and proliferation of PCa cells in vivo. (A) Representative images of the nude BALB/c mouse model of popliteal LN metastasis. The indicated PC3 cells were injected into the footpads of the nude mice, and the popliteal LNs were enucleated and analyzed. (B and C) Representative images of bioluminescence (popliteal LNs) (B) and quantification analysis of popliteal LN metastasis (C) in the indicated cell groups (n = 5 per group). (D and E) Representative images of dissected popliteal LNs (D) and quantification analysis (E) of the LN volume. (F) Representative images of H&E staining confirming the LN status (n = 5). The black arrow shows the PCa cell. (G and H) Representative images of the tumors of DEPDC1B‐knockdown (G) or ‐overexpression (H) groups and their respective controls. (I) Tumor growth curves of DEPDC1B‐knockdown (top panels) or ‐overexpression groups (bottom panels) are summarized in the line chart. The average tumor volume is expressed as the mean ± SD of five mice. (J) Tumor weights were measured after the tumors were surgically dissected. (K) The correlation of expression scores between Ki67 and DEPDC1B in xenograft tumors. (L) IHC examination of xenograft tumor DEPDC1B and Ki67 expression. *P < .05, **P < .01, and ***P<.001. Scale bars: 50 µm (red), 500 µm (black)

To explore the effects of DEPDC1B in PCa metastasis in vivo, a popliteal LN metastasis model was constructed in nude mice (Figure 3A). PC3/luciferase (PC3/luc) PCa cell lines with stable knockdown or overexpression of DEPDC1B were inoculated into the footpads of BALB/c nude mice. The primary footpad tumors were dissected with popliteal LNs after 8 weeks. Obviously, downregulating DEPDC1B significantly inhibited PCa cell metastasis to LNs. In contrast, overexpression of DEPDC1B promoted LN metastasis, as determined by bioluminescence imaging system. Moreover, the volumes of the popliteal LNs in DEPDC1B‐overexpressing mice were largest, while they were smallest in the DEPDC1B shRNA mice (Figures 3B‐E). H&E staining was also used to confirm PCa cell metastatic LNs (Figure 3F).

To further investigate the function of DEPDC1B in PCa tumorigenesis in vivo, stable DEPDC1B‐overexpressing, DEPDC1B‐silenced, or control PC3 cells were subcutaneously injected into BALB/c nude mice, and the features of the tumors were measured every 3 days. Strikingly, compared with the control group, the growth rate, size, and weight of tumors derived from the DEPDC1B‐knockdown group were significantly reduced. Conversely, DEPDC1B‐overexpression promoted the tumor growth of PCa cells (Figures 3G‐J). Moreover, the tumors derived from the DEPDC1B‐silencing group revealed lower expression of the proliferation marker Ki67 than the control group. However, the opposite results were found in the DEPDC1B‐upregulation group (Figure 3K and L). Taken together, DEPDC1B facilitates metastasis and tumor growth of PCa cells in vivo.

### 3.5 DEPDC1B interacts with Rac1 to enhance theRac1‐PAK1 signaling pathway

**FIGURE 4:**
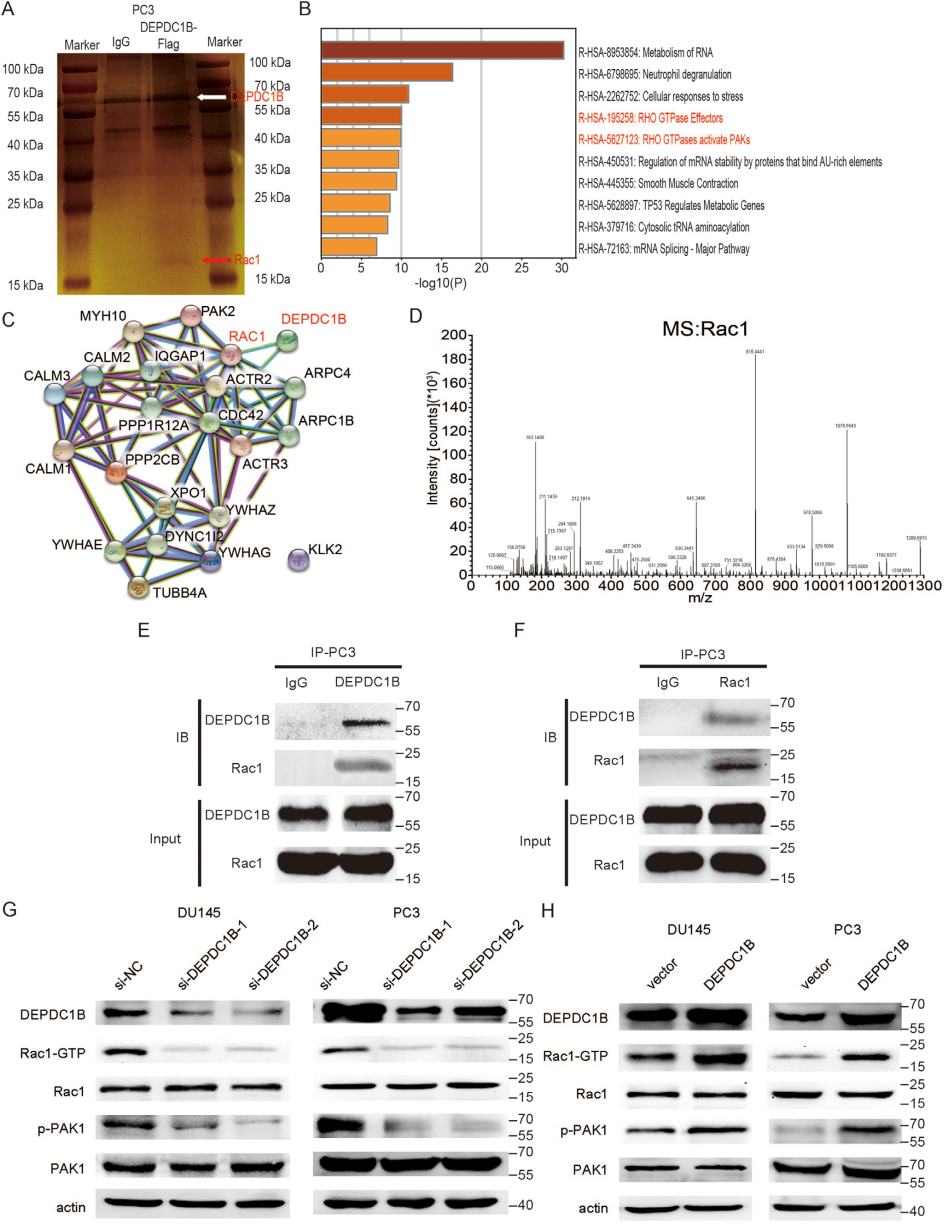
DEPDC1B regulates the Rho signaling pathway and binds to Rac1. (A) Representative image of silver‐stained SDS‐PAGE gels showing separated proteins that were pulled down using Flag‐labeled DEPDC1B. Anti‐IgG was used as the negative control. (B) The bar graph of top 10 nonredundant enrichment clusters of KEGG using the Metascape website. (C) PPI network visualization in String website showing the proteins that related to DEPDC1B and Rac1. (D) The mass spectrum of a representative peptide fragment of Rac1. (E and F) Western blot analysis determined that DEPDC1B is correlated with Rac1 after performing the pull‐down assay with Flag‐labeled DEPDC1B (E) and an anti‐Rac1 (F) immunoprecipitation antibody. Anti‐IgG was used as the negative control protein in the pull‐down assay. (G and H) Representative image of the Western blotting analysis of active Rac1, total Rac1, phosphorylated PAK1, total PAK1 protein levels after DEPDC1B‐knockdown (G) or ‐overexpression (H) in DU145 and PC3 cells

To explore the mechanism of DEPDC1B regulates the metastasis in PCa. We performed a coimmunoprecipitation (co‐IP) experiment using Flag‐labeled DEPDC1B as bait to identify DEPDC1B‐interacting proteins in PC3 cell line. The magnetic beads were collected for analysis by MS, and an obvious band with a molecular weight between 15 and 25 kDa was observed (Figure 4A). Several proteins were hit by MS, and we constructed an enrichment analysis and protein‐protein interaction (PPI) analysis by entering them into the Metascape website and String website. The results are shown in Figure 4B and C. Two of the top 10 enrichment pathways are related to the Rho family, which is related to cell migration, while PPI analysis showed that Rac1 is one of the members correlated with DEPDC1B. The mass spectrometer of Rac1 is shown in Figure 4D. To confirm the interaction between DEPDC1B and Rac1, the proteins that were pulled down by Flag‐labeled DEPDC1B were detected by the Rac1 antibody, and we found that DEPDC1B can interact with Rac1 (Figure 4E and Supporting information Figure S3B). Equivalently, the proteins that were pulled down by Rac1 could be detected by the DEPDC1B antibody (Figure 4F and Supporting information Figure S3C). These results indicated that DEPDC1B can bind to Rac1.

To further investigate the relationship between DEPDC1B and the Rac1 signaling pathway, we constructed a PAK‐PBD pull‐down assay to explore whether DEPDC1B can transfer Rac1‐GDP to Rac1‐GTP in PCa cells. We found that overexpressing DEPDC1B increased GTP loading in Rac1 proteins, while knockdown of DEPDC1B reduced the level of Rac1‐GTP (Figure 4G and H, Supporting information Figure S3A). Moreover, PAK1, downstream of Rac1, was phosphorylated when DEPDC1B was upregulated, and the level of phosphorylated PAK1 was reduced by DEPDC1B ablation (Figure 4G and H, Supporting information Figure S3A). The protein levels of total Rac1 and PAK1 remained the same regardless of how DEPDC1B changed. These results demonstrated that DEPDC1B was a potential activator of Rac1 and enhanced the Rac1‐PAK1 signaling pathway to promote PCa metastasis.

### 3.6 DEPDC1B facilitates PCa metastasis and proliferation through Rac1‐PAK1 pathway

**FIGURE 5:**
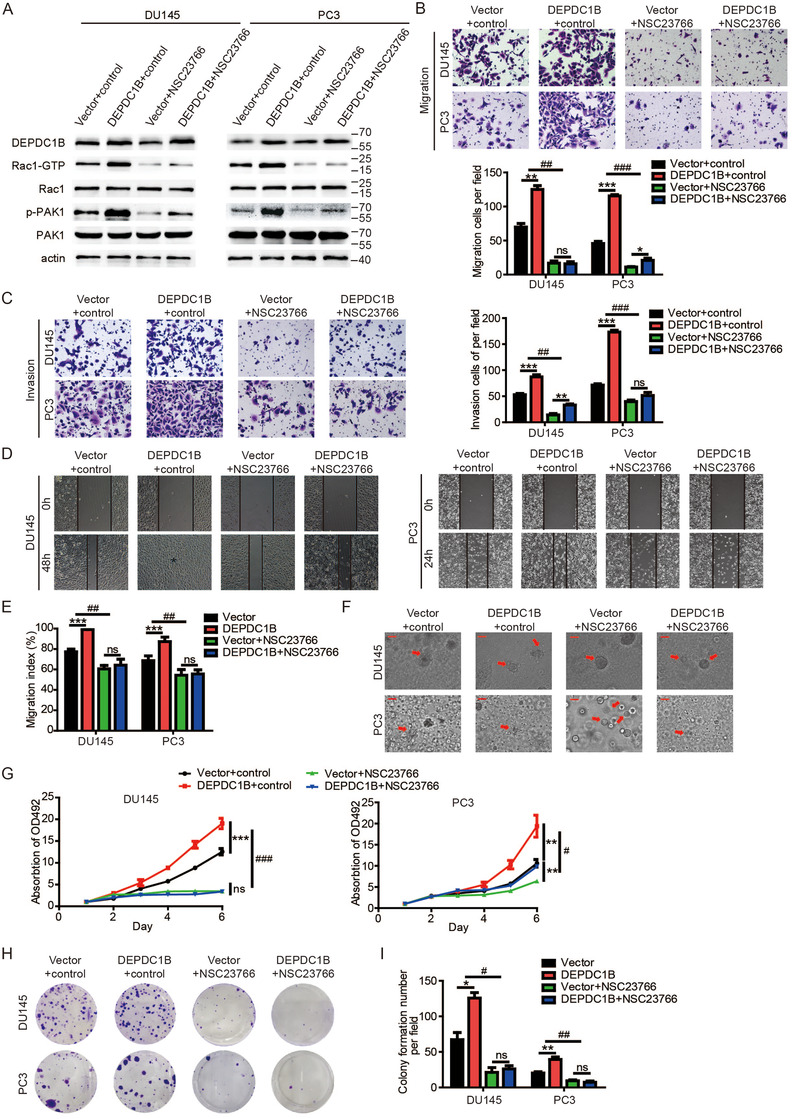
Inhibitor of active Rac1 (NSC23766) reverses the function in DEPDC1B‐overexpressing cells. (A) The levels of active Rac1, total Rac1, phosphorylated PAK1, and total PAK1 protein were detected by Western blotting in DEPDC1B‐overexpressing cells combined with NSC23766. (B and C) Representative images of cell migration (B) and invasion (C) were analyzed using DEPDC1B‐overexpressing or control cells combined with NSC23766 (left panels) and a quantification analysis of migrated or invaded cell counts (right panels). (D) Representative images of wound‐healing assays using DEPDC1B‐overexpressing or control cells combined with NSC23766 in DU145 (left panels) and PC3 (right panels) cells, showing reversing cell motility after using NSC23766 in DEPDC1B‐overexpressing cells. (E) A quantification analysis of cell migration index is shown. (F) Representative images of three‐dimensional (3D) cell culture using DEPDC1B‐overexpressing or control cells combined with NSC23766, showing reversing cell motility after using NSC23766 in DEPDC1B‐overexpressing cells. (G) Cell viability was reversed by using NSC23766 in DEPDC1B‐overexpressing or control cells. (H) Colony formation assays were constructed in DEPDC1B‐overexpressing or control cells combined with NSC23766. (I) A quantification analysis of colony formation number was shown. NSC23766 was used at 30 µM for 48 h. Unpaired *t*‐test was used to analyze two groups of data; *P < .05, **P < .01, and ***P<.001. Two‐way ANOVA was performed to analyze factorial designed data, # *P* < .05, ## *P* < .01, and ###*P*<.001. Scale bars: 25 µm

To explore whether DEPDC1B regulates PCa cell in Rac1 dependent manner, we overexpressed DEPDC1B and then used an inhibitor of Rac1‐GTP (NSC23766) to inhibit Rac1 specific activation and siRNAs to knockdown the expression of Rac1 in PCa cells. Then, we found that both NSC23766 and Rac1‐specific siRNA reversed DEPDC1B‐induced activation of Rac1‐GTP and phosphorylation of PAK1 (Figure 5A and Supporting information Figure S3A). Interestingly, reducing the level of Rac1‐GTP attenuated the DEPDC1B‐mediated role of the metastasis of PCa cells in vitro (Figure 5B‐F). As expected, the proliferation in PCa cells enhanced by DEPDC1B was also eliminated by NSC23766 (Figure 5G‐I). The similar results were found when we used Rac1‐specific siRNAs to suppress Rac1 expression in DEPDC1B‐upregulated PCa cells (Supporting information Figure S4).

**FIGURE 6:**
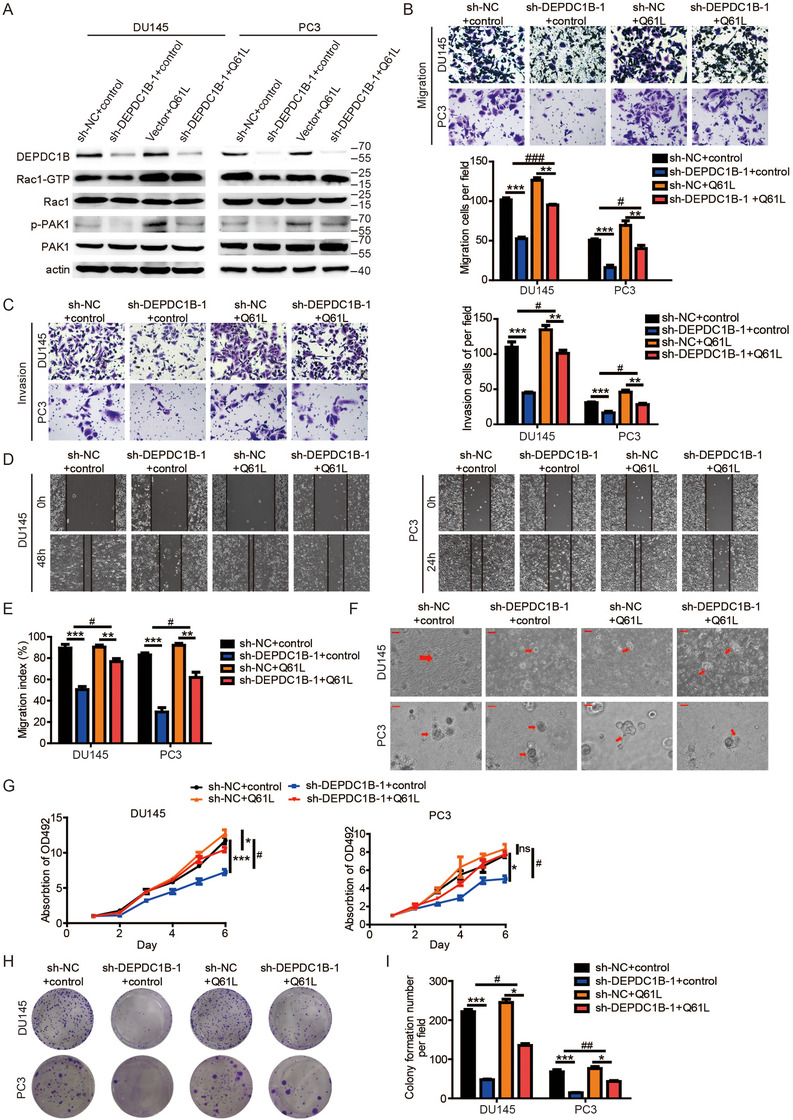
Mutated active Rac1 plasmid (Q61L) recuses the function in DEPDC1B‐knockdown cells. (A) The levels of active Rac1, total Rac1, phosphorylated PAK1, and total PAK1 protein were detected by Western blotting in DEPDC1B‐knockdown cells combined with Q61L. (B and C) Representative images of cell migration (B) and invasion (C) were analyzed using DEPDC1B‐knockdown or control cells combined with Q61L (left panels), and a quantification analysis of the migrated or invaded cell counts (right panels) is shown. (D) Representative images of wound‐healing assays using DEPDC1B‐knockdown or control cells combined with Q61L in DU145 (left panels) and PC3 (right panels) cells, showing reversing cell motility after using Q61L in DEPDC1B‐overexpressing cells. (E) A quantification analysis of cell migration index is shown. (F) Representative images of three‐dimensional (3D) cell culture using DEPDC1B‐knockdown or control cells combined with Q61L showing rescued cell motility after using Q61L in DEPDC1B‐knockdown cells. (G) Cell viability was rescued by using Q61L in DEPDC1B‐knockdown or control cells. (H) Colony formation assays were constructed in DEPDC1B‐knockdown or control cells combined with NSC23766. (I) A quantification analysis of the colony formation number was shown. Unpaired *t*‐test was used to analyze two groups of data; *P < .05, **P < .01, and ***P<.001. Two‐way ANOVA was performed to analyze factorial designed data, # *P* < .05, ##*P* < .01, and ###*P*<.001. Scale bars: 25 µm

To further address this, DEPDC1B knockdown PCa cells were transfected with the Rac1 Q61L plasmid (a constitutively active mutant of Rac1). The data showed that transfection of the Q61L plasmid into stable knockdown DEPDC1B PCa cells rescued DEPDC1B knockdown‐induced inhibition on the Rac1‐PAK1 signaling pathway (Figure 6A). The DEPDC1B knockdown‐induced inhibition of metastasis and proliferation in PCa cells was rescued by transfection with the Q61L plasmid (Figure 6B‐I). All the results indicated that DEPDC1B promoted metastasis and proliferation of PCa cell in Rac1‐dependent manner.

### 3.7 DEPDC1B promotes EMT to induce metastasis in PCa

**FIGURE 7:**
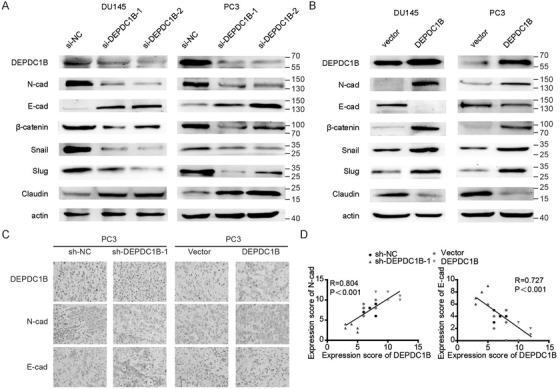
DEPDC1B promotes EMT to induce metastasis in PCa. (A and B) Representative image of the Western blotting analysis of N‐cadherin, E‐cadherin, β‐catenin, snail, slug, and claudin1 protein levels after DEPDC1B‐knockdown (A) or ‐overexpression (B) in DU145 and PC3 cells. (C) IHC examination of DEPDC1B, N‐cadherin, and E‐cadherin expression in xenograft tumors. (D) The correlation of expression scores between DEPDC1B and *N*‐cadherin (right panel) or E‐cadherin (left panel) in xenograft tumors

Accumulating evidences show that the Rac1‐PAK1 signaling pathway has been implicated in the process of cellular migration by inducing EMT,^27^, ^28^, ^29^ and EMT is one of the most important mechanisms in cancer metastasis.^30^, ^31^ To explore whether EMT is required for DEPDC1B‐induced PCa metastasis, we detected the EMT markers by western blotting after overexpressing or silencing DEPDC1B. The results revealed that DEPDC1B knockdown increased the levels of E‐cadherin and Claudin1 while reducing the levels of N‐cadherin, Snail, Slug, and β‐catenin, suggesting that the EMT process was inhibited (Figure 7A and Supporting information Figure S5A). And DEPDC1B overexpression‐induced EMT in both DU145 and PC3 cell lines (Figure 7B and Supporting information Figure S5A). Moreover, the DEPDC1B‐silencing group showed an increasing level of E‐cadherin and reduced level of N‐cadherin in the tumors, while the opposite results were found in the DEPDC1B‐upregulation group (Figure 7C‐D). These results suggest that DEPDC1B promotes EMT to induce metastasis in PCa.

### 3.8 DEPDC1B induces EMT in PCa via the Rac1‐PAK1 pathway

**FIGURE 8:**
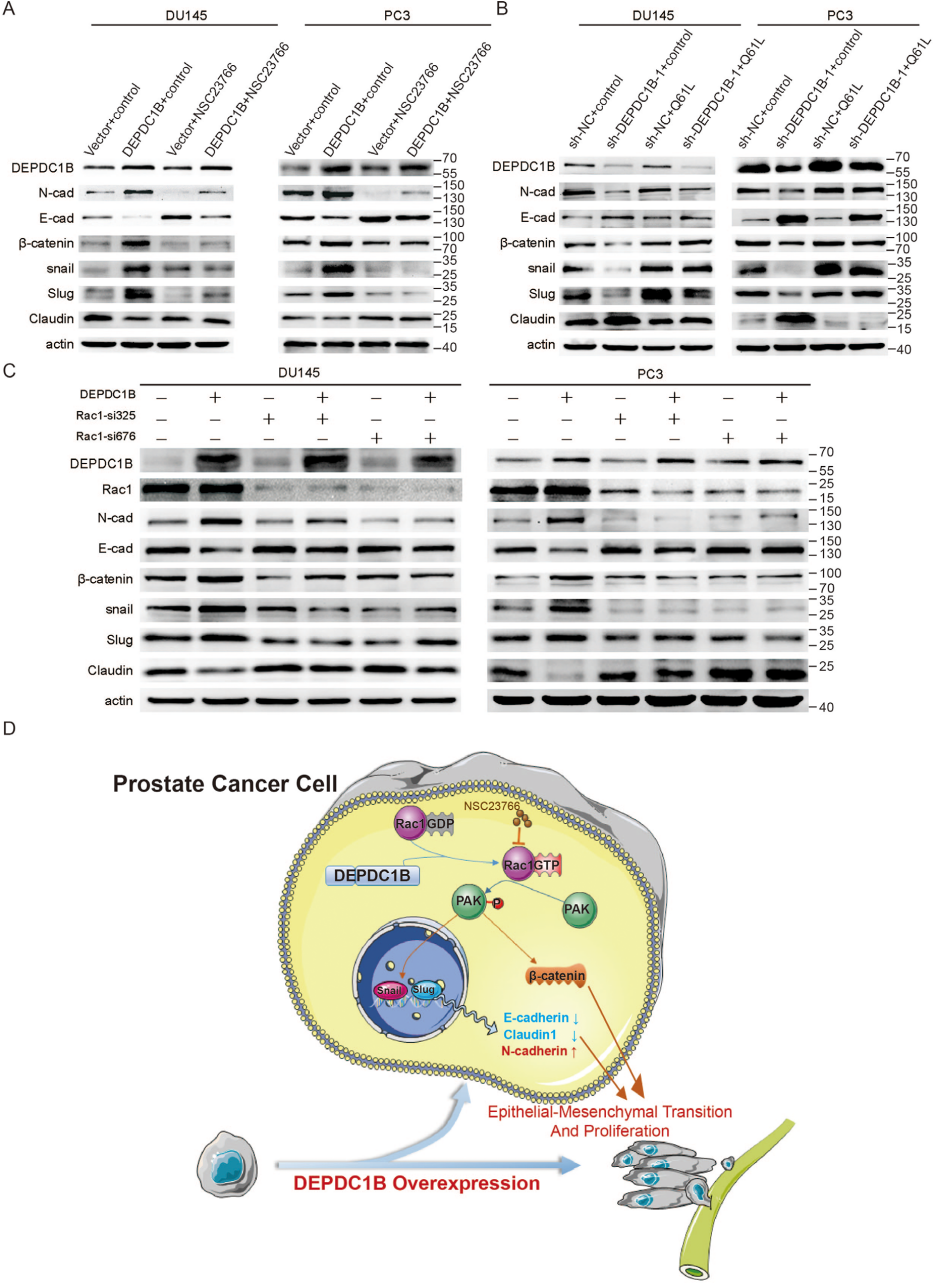
DEPDC1B induces EMT in PCa via the Rac1‐PAK1 pathway. (A) The levels of N‐cadherin, E‐cadherin, β‐catenin, snail, slug, and claudin1 protein were detected by Western blotting in DEPDC1B‐overexpressing cells combined with NSC23766. (B) The levels of N‐cadherin, E‐cadherin, β‐catenin, snail, slug, and claudin1 protein were detected by Western blotting in DEPDC1B‐knockdown cells combined with Q61L. (C) The levels of N‐cadherin, E‐cadherin, β‐catenin, snail, slug, and claudin1 protein were detected by Western blotting in DEPDC1B‐overexpressing cells combined with Rac1 iRNA. (D) A schematic model of the mechanism underlying the role of DEPDC1B in PCa metastasis and progression

To further explore the molecular mechanism underlying DEPDC1B‐induced metastasis and proliferation in PCa, we treated PCa cells stably overexpressing DEPDC1B with NSC23766‐specific or Rac1‐specific siRNA. After treatment with NSC23766‐specific or Rac1‐specific siRNAs, E‐cadherin, and claudin1 were increased while N‐cadherin, snail, slug, and β‐catenin were reduced in DEPDC1B‐overexpressing PCa cells, which indicated inhibition of the EMT process (Figure 8A and B). In contrast, transfection of the Q61L plasmid rescued the reduction in N‐cadherin, snail, slug, and β‐catenin, and the increase in E‐cadherin and Claudin1 induced by DEPDC1B knockdown (Figure 8C). Collectively, DEPDC1B induces EMT and enhances PCa cell migration and progression via the Rac1‐PAK1 signaling pathway.

## 4 DISCUSSION

Cancer metastasis is one of the most important causes of mortality and contributes to approximately 90% of cancer deaths, especially for PCa, for which the 5‐year survival rate is only 29% after metastasis.^1^, ^5^ Therefore, it is emergent to develop the novel effective treatment for mPCa.^32^ In the present study, we comprehensibly analyzed mutidatasets and found a protein called DEPDC1B correlated with mPCa. Overexpression of DEPDC1B correlated with high Gleason scores and high malignancy. Furthermore, we found that DEPDC1B plays a crucial role in mPCa and may be a potential treatment target for mPCa.

Previous studies showed that DEPDC1B played a key role in the GPCR and Rho signaling pathways.^12^, ^14^, ^15^ Here, we found that DEPDC1B enhanced the transformation from Rac1‐GDP into Rac1‐GTP and phosphorylate PAK1 by interacting with Rac1 and then activating the Rac1‐PAK1 signaling pathway. We hypothesized that the two domains of DEPDC1B may contribute to elevating the level of Rac1‐GTP. First, the DEP domain of DEPDC1B interacted with GPCRs and mediated GPCR signaling pathways. Moreover, it could interact with Rac1 to modulate its subcellular localization and recruit the GEFs of Rac1 to transform Rac1‐GDP to Rac1‐GTP.^13^, ^14^ Second, the inactive RhoGAPs domain of DEPDC1B might prevent the deactivation Rac1‐GTP from other GAPs. The ruffling of membrane, a characteristic feature of many actively migrating cells, was controlled by a group of enzymes, including Rac1, which regulate it by modulating the polymerization of actin at the leading edge.^33^ DEPDC1B also influences the formation of focal adhesions to regulate the mortality of cells.^16^ Therefore, our data suggested that DEPDC1B might also regulate the ruffling of membrane to promote metastasis via Rac1‐PAK1 signaling in PCa. Ying‐Fang Su et al indicated that DEPDC1B is able to modulate Rac1 cellular localization in rat embryonic fibroblasts and enhance anchorage‐independent growth by activating the Rac1‐ERK signaling pathway.^13^ Our study found that DEPDC1B plays a different role in regulating PCa cell migration and proliferation by inducing EMT via the Rac1‐PAK1 signaling pathway.

EMT is a gradual cellular process with several states that plays a key role in cancer metastasis.^30^, ^34^ A series of heterotypic cell‐cell signaling molecules, such as the Wnt, TGFβ, and Notch signaling molecules, are involved in inducing EMT.^35^ In general, epithelial markers, such as E‐cadherin, decrease while mesenchymal markers increase during EMT. However, the variation in these different markers is not the same in the different EMT states.^30^, ^35^ In our study, we found that overexpressed DEPDC1B decreased the expression of E‐cadherin and Claudin1, while increased the expression of N‐cadherin, snail, and slug in PCa cells. Interestingly, we also found that the level of β‐catenin decreased when DEPDC1B was knocked down. A previous study found that PAK1 can stabilize β‐catenin by phosphorylating it, making the transcription of β‐catenin more active.^36^ To summarize, DEPDC1B regulates the mobility of PCa by inducing EMT and stabilizing Wnt/β‐catenin signaling by activating the Rac1‐PAK1 pathway.

Additionally, we also found that DEPDC1B ablation also inhibited the proliferation of PCa and that the function was also regulated by DEPDC1B via Rac1‐PAK1 signaling. Several studies have demonstrated that Rac1‐PAK1 signaling can regulate the proliferation of cells via multiple proteins such as Cyclin D1 and IL‐2.^37^, ^38^, ^39^, ^40^ In addition, DEPDC1B might also influence proliferation by inducing apoptosis or autophagy via modulating Wnt/β‐catenin signaling.^41^, ^42^

## 5 CONCLUSIONS

It is our novel discovery that DEPDC1B induces EMT and promotes the mobility and proliferation of PCa via Rac1‐PAK1 signaling pathway by interacting with Rac1.Therefore, our findings provide insight into DEPDC1B and indicate that Rac1 might be an important treatment target against mPCa (Figure 8D).

## ETHICS APPROVAL AND CONSENT TO PARTICIPATE

We obtained human prostate samples by surgery, needle biopsy or transurethral resection prostate (TURP) with the written consent of patients who underwent surgery at Sun Yat‐sen University Cancer Center. Ethical consent was approved by Sun Yat‐sen University's Committees for Ethical Review of Research involving Human Subjects.

## CONFLICT OF INTEREST

The authors declare no competing interests.

## AUTHOR CONTRIBUTIONS

Hai Huang, Xu Chen designed the study, analyzed data, and performed the initial experimental design. Zean Li and Xu Chen wrote the manuscript, performed data analysis. Xu Chen, Fen Wang, and Yiming Lai critically revise the draft for important intellectual content. Zean Li, Qiong Wang, and Shirong Peng participated in the experiment, performed the immunohistochemistry (IHC) experiments. Shirong Peng, Kaiwen Li analyzed the clinical data. Yiran Tao and Junxiu Chen performed the experiment for cell viability. WanHuaWu and ZeGao performed the experiment for qPCR and western blot. All authors read and approved the final manuscript.

## Supporting information

Supporting information

## Data Availability

Three datasets (GSE3325, GSE6919, and GSE67872) analyzed during the current study are available in the Gene Expression Omnibus, https://www.ncbi.nlm.nih.gov/geo/.

## References

[1] Bray F , Ferlay J , Soerjomataram I , et al. Global cancer statistics 2018: gLOBOCAN estimates of incidence and mortality worldwide for 36 cancers in 185 countries. CA Cancer J Clin. 2018;68(6):394‐424. 30207593 10.3322/caac.21492

[2] Li X , Zhu J , Liu Y , et al. MicroRNA‐331‐3p inhibits epithelial‐mesenchymal transition by targeting ErbB2 and VAV2 through the Rac1/PAK1/β‐catenin axis in non‐small‐cell lung cancer. Cancer Sci. 2019;110:1883‐1896. 30955235 10.1111/cas.14014 PMC6550127

[3] Siegel RL , Miller KD , Jemal A . Cancer satistics, 2017. CA Cancer J Clin. 2017;67(1):7‐30. 28055103 10.3322/caac.21387

[4] Li ZA , Li K , Wu W , et al. The comparison of transurethral versus suprapubic catheter after robot‐assisted radical prostatectomy: a systematic review and meta‐analysis. Transl Androl Urol. 2019;8(5):476‐488. 31807425 10.21037/tau.2019.08.25 PMC6842775

[5] Guan X . Cancer metastases: challenges and opportunities. Acta Pharm Sin B. 2015;5(5):402‐418. 26579471 10.1016/j.apsb.2015.07.005 PMC4629446

[6] Pradhan AK , Bhoopathi P , Talukdar S , et al. Recombinant MDA‐7/IL24 suppresses prostate cancer bone metastasis through downregulation of the Akt/Mcl‐1Pathway. Mol Cancer Ther. 2018;17(9):1951‐1960. 29934341 10.1158/1535-7163.MCT-17-1002 PMC7598934

[7] Gundem G , Van Loo P , Kremeyer B , et al. The evolutionary history of lethal metastatic prostate cancer. Nature. 2015;520(7547):353‐357. 25830880 10.1038/nature14347 PMC4413032

[8] Jaffe AB , Hall A . Rho GTPases: biochemistry and biology. Annu Rev Cell Dev Biol. 2005;21:247‐269. 16212495 10.1146/annurev.cellbio.21.020604.150721

[9] Baker MJ , Abba MC , Garcia‐Mata R , Kazanietz MG . P‐REX1‐Independent, calcium‐dependent RAC1 hyperactivation in prostate cancer. Cancers (Basel). 2020;12(2):480. 10.3390/cancers12020480 PMC7072377 32092966

[10] Dirat B , Ader I , Golzio M , et al. Inhibition of the GTPase Rac1 mediates the antimigratory effects of metformin in prostate cancer cells. Mol Cancer Ther. 2015;14(2):586‐596. 25527635 10.1158/1535-7163.MCT-14-0102

[11] Tajadura‐Ortega V , Garg R , Allen R , et al. An RNAi screen of Rho signalling networks identifies RhoH as a regulator of Rac1 in prostate cancer cell migration. Bmc Biol. 2018;16(1):29. 29510700 10.1186/s12915-018-0489-4 PMC5840776

[12] Yang Y , Liu L , Cai J , et al. DEPDC1B enhances migration and invasion of non‐small cell lung cancer cells via activating Wnt/β‐catenin signaling. Biochem Bioph Res Co. 2014;450(1):899‐905. 10.1016/j.bbrc.2014.06.076 24971537

[13] Su Y , Liang C , Huang C , et al. A putative novel protein, DEPDC1B, is overexpressed in oral cancer patients, and enhanced anchorage‐independent growth in oral cancer cells that is mediated by Rac1 and ERK. J Biomed Sci. 2014;21(1):67. 25091805 10.1186/s12929-014-0067-1 PMC4237807

[14] Ballon DR , Flanary PL , Gladue DP , et al. DEP‐domain‐mediated regulation of GPCR signaling responses. Cell. 2006;126(6):1079‐1093. 16990133 10.1016/j.cell.2006.07.030

[15] Bos JL , Rehmann H , Wittinghofer A . GEFs and GAPs: critical elements in the control of small G proteins. Cell. 2007;129(5):865‐877. 17540168 10.1016/j.cell.2007.05.018

[16] Garcia‐Mata R . Arrested detachment: a DEPDC1B‐mediated de‐adhesion mitotic checkpoint. Dev Cell. 2014;31(4):387‐389. 25458006 10.1016/j.devcel.2014.11.008

[17] Marchesi S , Montani F , Deflorian G , et al. DEPDC1B coordinates de‐adhesion events and cell‐cycle progression at mitosis. Dev Cell. 2014;31(4):420‐433. 25458010 10.1016/j.devcel.2014.09.009 PMC4250264

[18] Bai S , Chen T , Du T , et al. High levels of DEPDC1B predict shorter biochemical recurrence‐free survival of patients with prostate cancer. Oncol Lett. 2017;14(6):6801‐6808. 29163701 10.3892/ol.2017.7027 PMC5686524

[19] Varambally S , Yu J , Laxman B , et al. Integrative genomic and proteomic analysis of prostate cancer reveals signatures of metastatic progression. Cancer Cell. 2005;8(5):393‐406. 16286247 10.1016/j.ccr.2005.10.001

[20] Chandran UR , Ma C , Dhir R , et al. Gene expression profiles of prostate cancer reveal involvement of multiple molecular pathways in the metastatic process. BMC Cancer. 2007;7:64. 17430594 10.1186/1471-2407-7-64 PMC1865555

[21] Ruscetti M , Dadashian EL , Guo W , et al. HDAC inhibition impedes epithelial‐mesenchymal plasticity and suppresses metastatic, castration‐resistant prostate cancer. Oncogene. 2016;35(29):3781‐3795. 26640144 10.1038/onc.2015.444 PMC4896852

[22] Gu P , Chen X , Xie R , et al. A novel AR translational regulator lncRNA LBCS inhibits castration resistance of prostate cancer. Mol Cancer. 2019;18(1):109. 31221168 10.1186/s12943-019-1037-8 PMC6585145

[23] Chen X , Xie R , Gu P , et al. Long noncoding RNA LBCS inhibits self‐renewal and chemoresistance of bladder cancer stem cells through epigenetic silencing of SOX2. Clin Cancer Res. 2019;25(4):1389‐1403. 30397178 10.1158/1078-0432.CCR-18-1656

[24] Chen Z , Chen X , Xie R , et al. DANCR promotes metastasis and proliferation in bladder cancer cells by enhancing IL‐11‐STAT3 signaling and CCND1 expression. Mol Ther. 2019;27(2):326‐341. 30660488 10.1016/j.ymthe.2018.12.015 PMC6391591

[25] Xie R , Chen X , Chen Z , et al. Polypyrimidine tract binding protein 1 promotes lymphatic metastasis and proliferation of bladder cancer via alternative splicing of MEIS2 and PKM. Cancer Lett. 2019;449:31‐44. 30742945 10.1016/j.canlet.2019.01.041

[26] Chen X , Zhang J , Ruan W , etal. Urine DNA methylation assay enables early detection and recurrence monitoring for bladder cancer. J Clin Invest. 2020 10.1172/jci139597. PMC7685755 32817589

[27] Li X , Zhu J , Liu Y , et al. MicroRNA‐331‐3p inhibits epithelial‐mesenchymal transition by targeting ErbB2 and VAV2 through the Rac1/PAK1/β‐catenin axis in non‐small‐cell lung cancer. Cancer Sci. 2019;110:1883‐1896. 30955235 10.1111/cas.14014 PMC6550127

[28] Cao F , Yin L . PAK1 promotes proliferation, migration and invasion of hepatocellular carcinoma by facilitating EMT via directly up‐regulating Snail. Genomics. 2019;112:694‐702. 31071459 10.1016/j.ygeno.2019.05.002

[29] Lv Z , Hu M , Zhen J , et al. Rac1/PAK1 signaling promotes epithelial‐mesenchymal transition of podocytes in vitro via triggering β‐catenin transcriptional activity under high glucose conditions. Int J Biochem Cell Biol. 2013;45(2):255‐264. 23153508 10.1016/j.biocel.2012.11.003

[30] Pastushenko I , Blanpain C . EMT transition states during tumor progression and metastasis. Trends Cell Biol. 2019;29(3):212‐226. 30594349 10.1016/j.tcb.2018.12.001

[31] Chaffer CL , San Juan BP , Lim E , Weinberg RA . EMT, cell plasticity and metastasis. Cancer Metast Rev. 2016;35(4):645‐654. 10.1007/s10555-016-9648-7 27878502

[32] Kelly SP , Anderson WF , Rosenberg PS , Cook MB . Past, current, and future incidence rates and burden of metastatic prostate cancer in the United States. Eur Urol Focus. 2018;4(1):121‐127. 29162421 10.1016/j.euf.2017.10.014 PMC6217835

[33] Nobes CD , Hall A . Rho, Rac, and Cdc42 GTPases regulate the assembly of multimolecular focal complexes associated with actin stress fibers, lamellipodia, and filopodia. Cell. 1995;81(1):53‐62. 7536630 10.1016/0092-8674(95)90370-4

[34] Heerboth S , Housman G , Leary M , et al. EMT and tumor metastasis. Clin Transl Med. 2015;4(1):1–13. 25852822 10.1186/s40169-015-0048-3 PMC4385028

[35] Chaffer CL , San JB , Lim E , Weinberg RA . EMT, cell plasticity and metastasis. Cancer Metastasis Rev. 2016;35(4):645‐654. 27878502 10.1007/s10555-016-9648-7

[36] Zhu G , Wang Y , Huang B , et al. A Rac1/PAK1 cascade controls beta‐catenin activation in colon cancer cells. Oncogene. 2012;31(8):1001‐1012. 21822311 10.1038/onc.2011.294

[37] Balasenthil S , Barnes CJ , Rayala SK , Kumar R . Estrogen receptor activation at serine 305 is sufficient to upregulate cyclin D1 in breast cancer cells. Febs Lett. 2004;567(2‐3):243‐247. 15178330 10.1016/j.febslet.2004.04.071

[38] Tao J , Oladimeji P , Rider L , Diakonova M . PAK1‐Nck regulates cyclin D1 promoter activity in response to prolactin. Mol Endocrinol. 2011;25(9):1565‐1578. 21719533 10.1210/me.2011-0062 PMC3165915

[39] Zhang J , Wang J , Zhou Y , et al. Rich1 negatively regulates the epithelial cell cycle, proliferation and adhesion by CDC42/RAC1‐PAK1‐Erk1/2 pathway. Cell Signal. 2015;27(9):1703‐1712. 26004135 10.1016/j.cellsig.2015.05.009

[40] Meyer ZBU , Brandenstein LI , von Elsner L , et al. RIT1 controls actin dynamics via complex formation with RAC1/CDC42 and PAK1. Plos Genet. 2018;14(5):e1007370. 29734338 10.1371/journal.pgen.1007370 PMC5937737

[41] Chang HW , Lee YS , Nam HY , et al. Knockdown of β‐catenin controls both apoptotic and autophagic cell death through LKB1/AMPK signaling in head and neck squamous cell carcinoma cell lines. Cell Signal. 2013;25(4):839‐847. 23280187 10.1016/j.cellsig.2012.12.020

[42] Anastas JN , Moon RT . WNT signalling pathways as therapeutic targets in cancer. Nat Rev Cancer. 2013;13(1):11‐26. 23258168 10.1038/nrc3419

