# Supplementary material for: The metastatic promoter DEPDC1B induces epithelial‐mesenchymal transition and promotes prostate cancer cell proliferation via Rac1‐PAK1 signaling
Source: Clin Transl Med. 2020 Oct 6;10(6):e191. doi: 10.1002/ctm2.191 (PMC7536616; doi:10.1002/ctm2.191)
Supplement: Supplementary file 1 — Supporting information [file CTM2-10-e191-s001.docx]

**Table S1 The primer, RNAi and shRNA sequences used in this article.**

| **Primer** |  |  |  | |
| --- | --- | --- | --- | --- |
| DEPDC1B | Forward | GAGCTACCAGGCTGTGGAAT | | |
|  | Reverse | GCCGAAGTTTTGACTGCACC | | |
| Rac1 | Forward | AACCAATGCATTTCCTGGAG | | |
|  | Reverse | TGTTTGCGGATAGGATAGGG | | |
| GAPDH | Forward | CAAGGCTGAGAACGGGAAG | | |
|  | Reverse | TGAAGACGCCAGTGGACTC | | |
|  |  |  |  | |
| **siRNA** |  | **Sequence 5'-3'** |  | |
| Si-NC | | UUCUCCGAACGUGUCACGUTT | |  |
| Si-DEPDC1B-1 | | GAUGCCGUUACGGAAACAUTT | |  |
| Si-DEPDC1B-2 | | CACAAGAGAACAUCCCAGUTT | |  |
| Si-Rac1-1 | | CCUGGAGAAUAUAUCCCUATT | |  |
| Si-Rac1-2 | | CUAAGGAGAUUGGUGCUGUTT | |  |
|  |  |  |  | |
| **shRNA** |  | **Sequence 5'-3'** |  | |
| Sh-NC | | UUCUCCGAACGUGUCACGUTT | |  |
| Sh-DEPDC1B-1 | | GAUGCCGUUACGGAAACAUTT | |  |

**Table S2 Associations between DEPDC1B expression and clinicopathological characteristics of PCa patient in Cohort 1.**

| **Clinical feature** | **Total patients n.** | **Low n.** | **High n.** | **P-value** |
| --- | --- | --- | --- | --- |
| **Type** | | | | |
| Normal | 32 | 21 | 11 | **0.033** |
| Tumor | 160 | 71 | 89 |  |
| **Age** | | | | |
| ≤65 | 77 | 35 | 42 | 0.559 |
| >65 | 116 | 58 | 58 |  |
| **Gleason score** | | | | |
| ≤6 | 18 | 13 | 5 | **＜0.001** |
| 3+4 | 28 | 21 | 7 |  |
| 4+3 | 10 | 4 | 6 |  |
| ≥8 | 104 | 33 | 71 |  |
| **T state** | | | | |
| T1-2 | 72 | 37 | 35 | 0.256 |
| T3-4 | 46 | 18 | 28 |  |
| **Lymph node metastasis** | | | | |
| N0 | 112 | 54 | 58 | 0.213 |
| N1 | 6 | 1 | 5 |  |
| **State** | | | | |
| ≤Ⅱb | 72 | 37 | 35 | 0.256 |
| ＞Ⅱb | 46 | 18 | 28 |  |

**Table S3 Associations between DEPDC1B expression and clinicopathological characteristics of PCa patients in TCGA database.**

| **Clinical feature** | **Total patients n.** | **Low n. (%)** | **High n. (%)** | **P-value** |
| --- | --- | --- | --- | --- |
| **Age** | | | | |
| ≤65 | 346 | 180 | 166 | 0.16 |
| >65 | 139 | 62 | 77 |  |
| **Gleason score** | | | | |
| ≤6 | 29 | 20 | 9 | **0.002** |
| 3+4 | 14 | 9 | 5 |  |
| 4+3 | 12 | 6 | 6 |  |
| ≥8 | 47 | 13 | 34 |  |
| **T state** | | | | |
| T1-2 | 186 | 126 | 60 | **＜0.001** |
| T3-4 | 292 | 111 | 181 |  |
| **Lymph node metastasis** | | | | |
| N0 | 336 | 163 | 173 | **0.022** |
| N1 | 77 | 26 | 51 |  |
| **Distant metastasis** | | | | |
| M0 | 444 | 221 | 223 | 0.248 |
| M1 | 3 | 0 | 3 |  |

**Supplemental Figure and Legend**


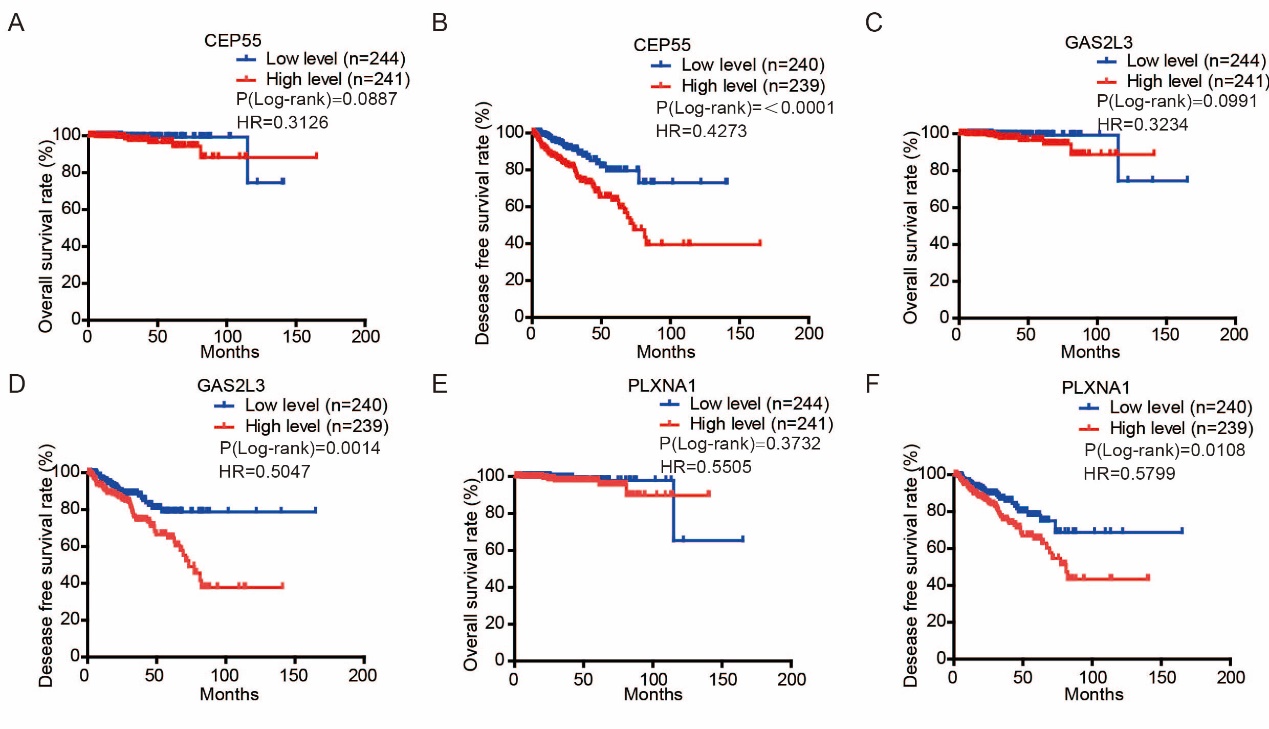


**Figure S1**

**The relationship between the expression of metastasis related genes and prognosis in PCa.**

(A-H) Kaplan-Meier curves for OS and DFS of PCa patients with high versus low expression of CEP55 (A and B), GAS2L3 (C and D) or PLXNA1 (E and F) in TCGA.

**
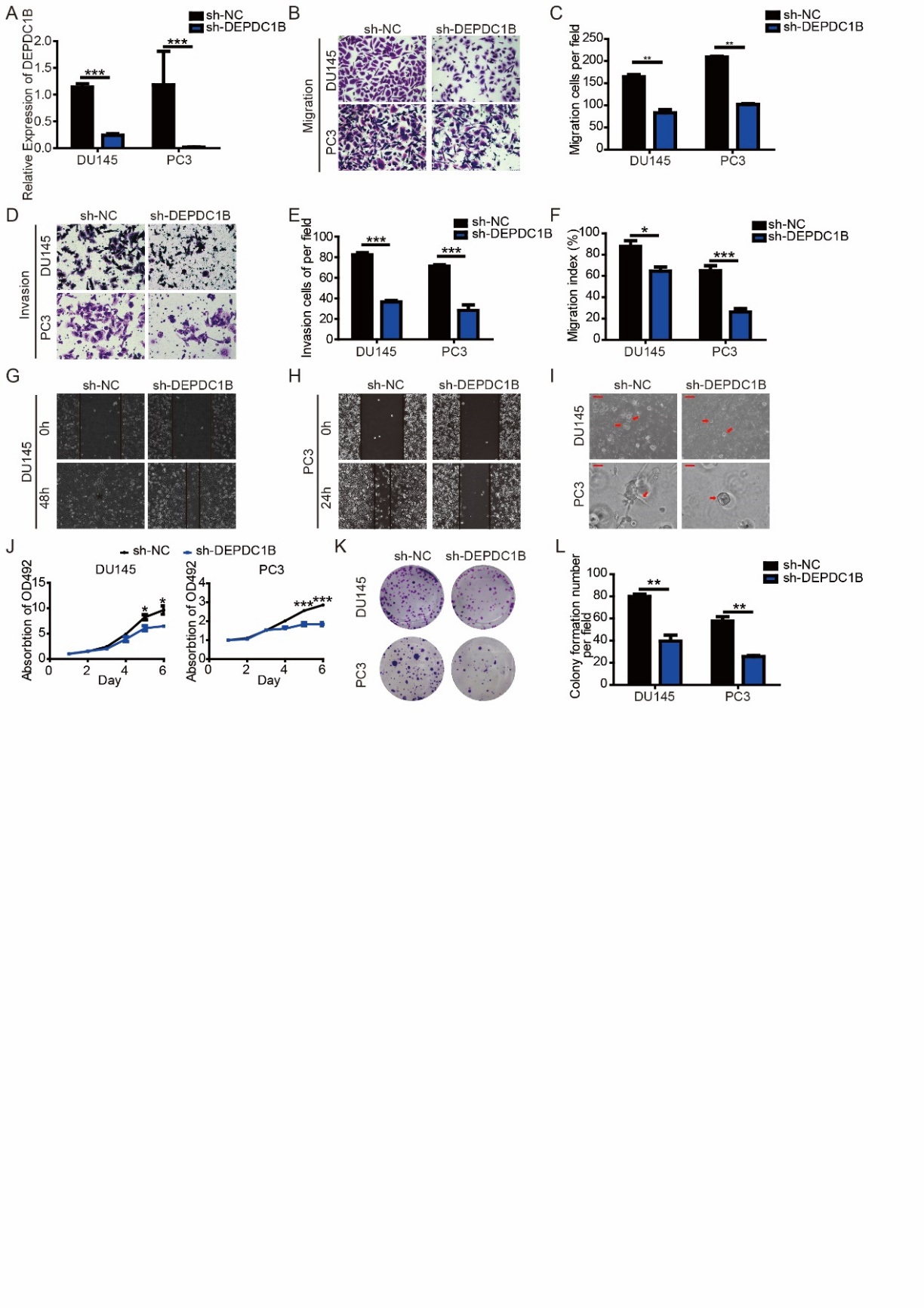
**

**Figure S2**

**Downregulating DEPDC1B suppresses the ability of metastasis and progression in PCa cells *in vitro*.**

(A) qRT-PCR analysis of DEPDC1B expression levels in stabled DEPDC1B-downregulated cells and control cells. (B-E) Representative images of migration (B) and invasion (D) assays using DU145 and PC3 cells, and a histogram analysis of migrated or invaded cell counts (C and E), showing cell migration and invasion after downregulation of DEPDC1B. (F) A histogram analysis of cell migration index is shown. (G-H) Representative images of wound-healing assays using DU145 (G) and PC3 (H) cells, showing cell motility after downregulation of DEPDC1B. (I) Representative images of three-dimensional (3D) cell culture using DU145 and PC3 cells, showing the cell invasive abilities in stereoscopic space. (J) Cell viability was evaluated in stabled DEPDC1B-downregulated DU145 and PC3 cells. (K) Colony formation assays were constructed in stabled DEPDC1B-downregulation DU145 and PC3 cells. (L) A histogram analysis of colony formation number was shown. *p < 0.05, **p < 0.01 and ***p＜0.001. Scale bars: 25 μm.


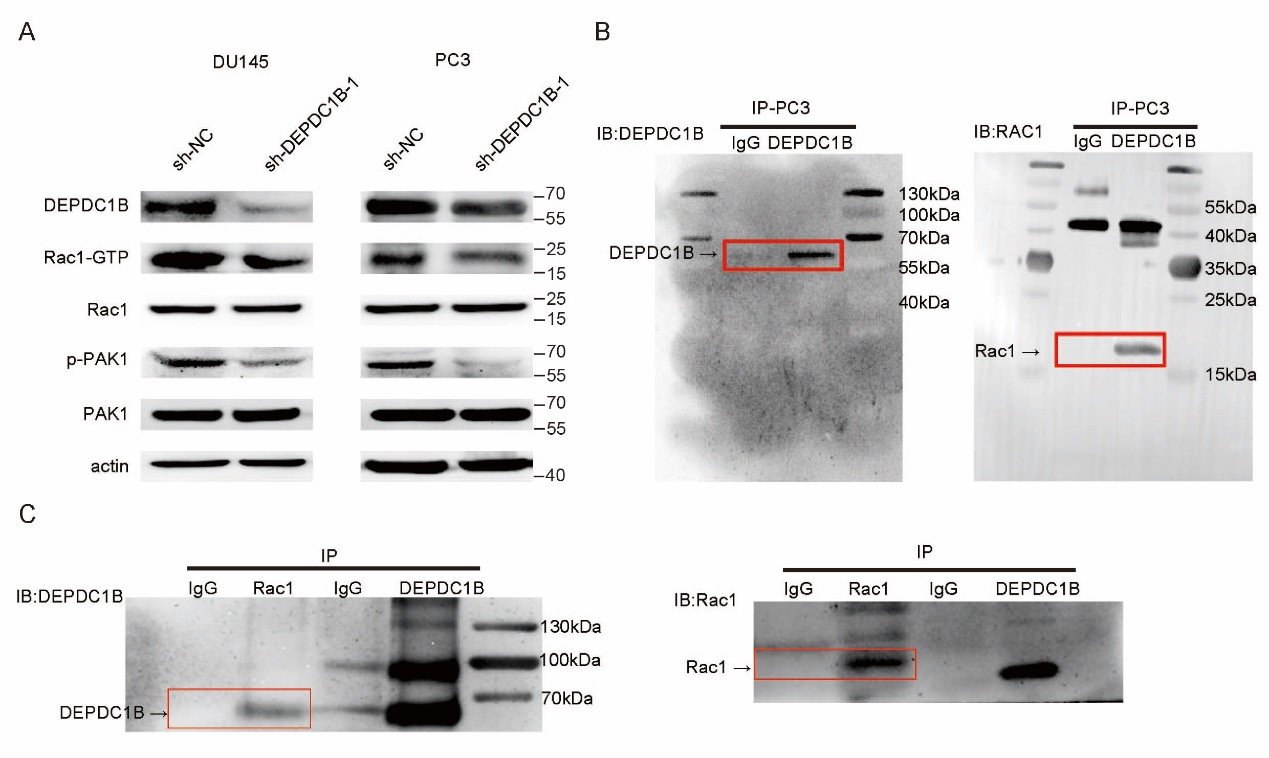


**Figure S3**

**DEPDC1B knockdown suppresses the activation of the Rac1-PAK1 signaling pathway** **and inhibited PCa EMT.**

(A) Representative image of the Western blotting analysis of active Rac1, total Rac1, phosphorylated PAK1, and the total PAK1 protein levels in stable DEPDC1B-downregulated DU145 and PC3 cells. (B and C) Full western blot for the pull-down assay with Flag-labeled DEPDC1B (B) and an anti-Rac1 (C) immunoprecipitation antibody with markers. Anti-IgG was used as the negative control protein in the pull-down assay. The images in the red box were shown in Figure 4E and 4F.

**
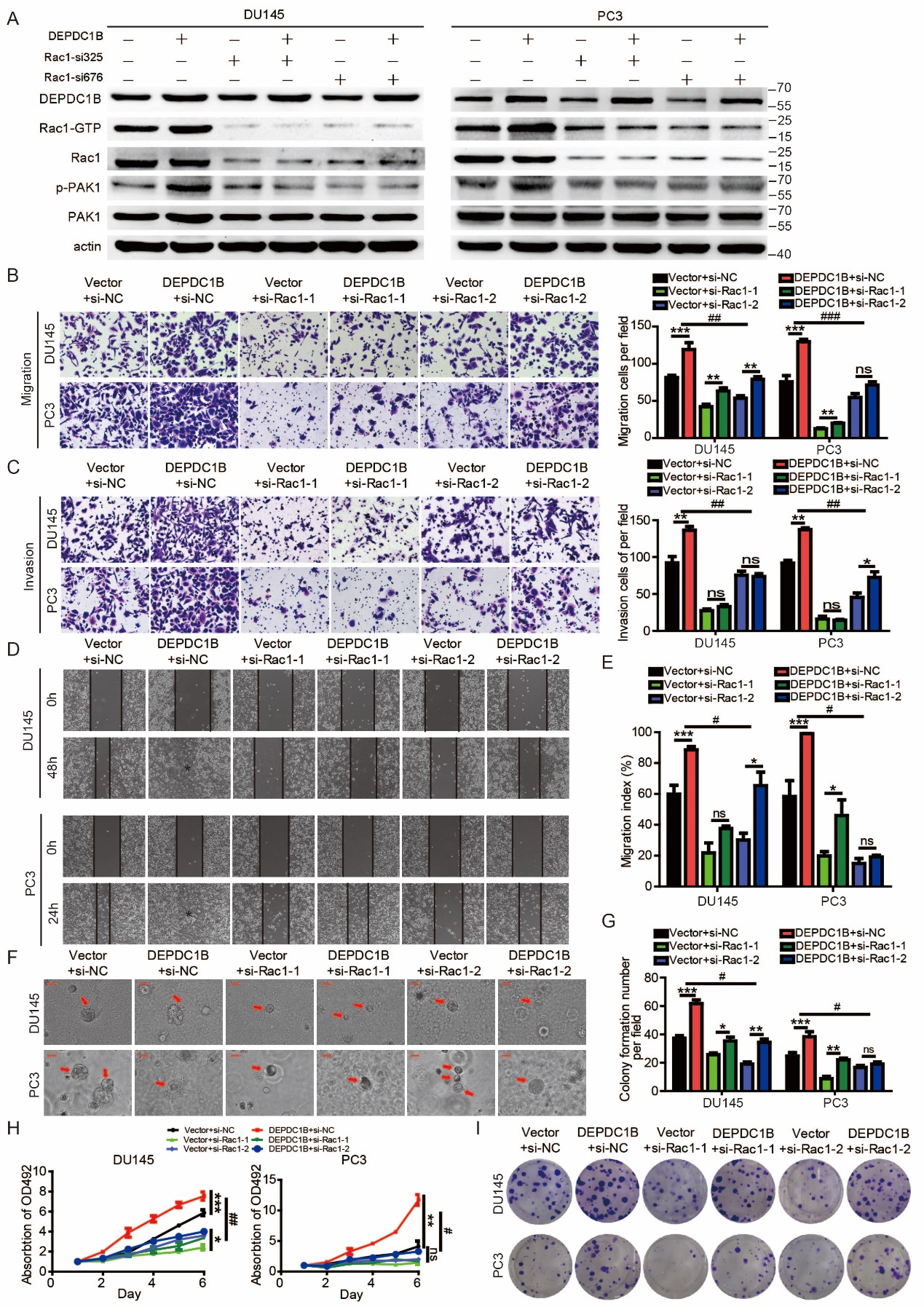
**

**Figure S4**

**Rac1 siRNA reverses the function in DEPDC1B-overexpressing cells.**

(A) The levels of active Rac1, total Rac1, phosphorylated PAK1, and total PAK1 protein were detected by Western blotting in DEPDC1B-overexpressing cells combined with Rac1 siRNA. (B and C) Representative images of cell migration (B) and invasion (C) were analyzed using DEPDC1B-overexpressing or control cells combined with Rac1 siRNA (left panels) and a histogram analysis of migrated or invaded cell counts (right panels). (D) Representative images of wound-healing assays using DEPDC1B-overexpressing or control cells combined with Rac1 siRNA in DU145 and PC3 cells, showing reversing cell motility after using Rac1 siRNA in DEPDC1B-overexpressing cells. (E) A histogram analysis of cell migration index is shown. (F) Representative images of three-dimensional (3D) cell culture using DEPDC1B-overexpressing or control cells combined with Rac1 siRNA, showing reversing cell motility after using Rac1 siRNA in DEPDC1B-overexpressing cells. (G) Cell viability was reversed by using Rac1 siRNA in DEPDC1B-overexpressing or control cells. (H) A histogram analysis of colony formation number was shown. (I) Colony formation assays were constructed in DEPDC1B-overexpressing or control cells combined with Rac1 siRNA. Unpaired t-test was used to analyze two groups of data; *p < 0.05, **p < 0.01 and ***p＜0.001. Two-way ANOVA was performed to analyze factorial designed data; # p < 0.05, ## p < 0.01 and ### p＜0.001. Scale bars: 25 μm.

**
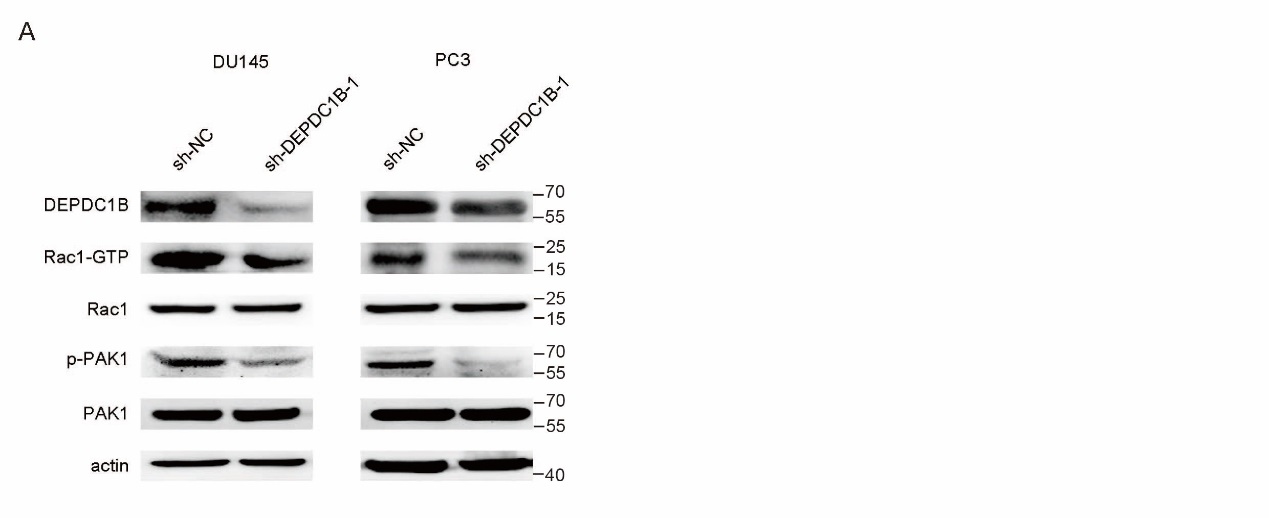
**

**Figure S5**

**DEPDC1B knockdown inhibits PCa EMT.**

(A) Representative image of the Western blotting analysis of N-cadherin, E-cadherin, β-catenin, snail, slug and claudin1 protein levels in stable DEPDC1B-downregulated DU145 and PC3 cells.
